# Supplementary material for: Controlling the pressure of hydrogen-natural gas mixture in an inclined pipeline
Source: PLoS One. 2020 Feb 27;15(2):e0228955. doi: 10.1371/journal.pone.0228955 (PMC7046196; doi:10.1371/journal.pone.0228955)
Supplement: S1 Fig — (PDF) [file pone.0228955.s001.pdf]

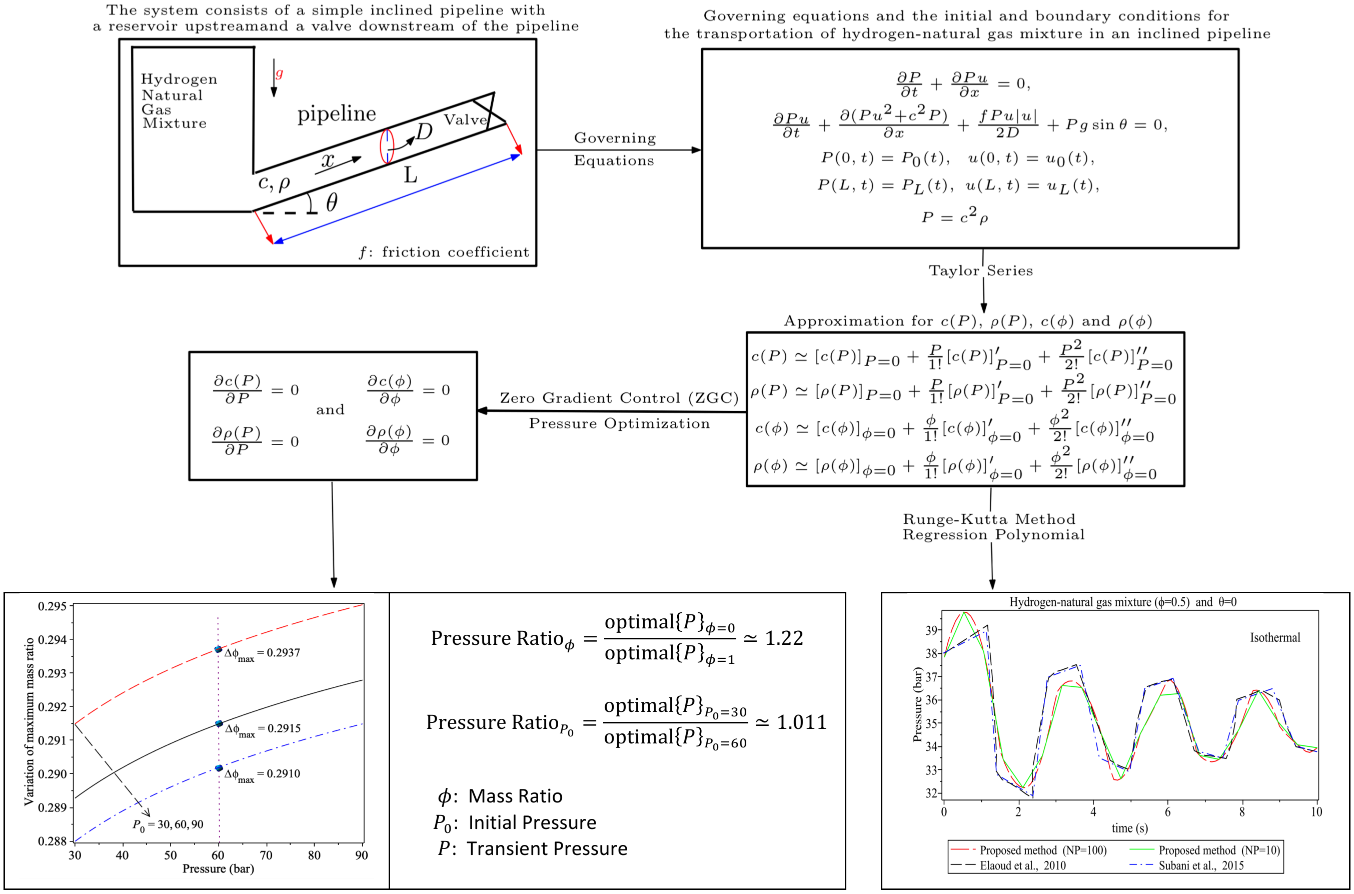

The system consists of a simple inclined pipeline with a reservoir upstreamand a valve downstream of the pipeline
Hydrogen Natural Gas Mixture
g
pipeline
Valve
D
x
L
c, ρ
θ
f: friction coefficient
Governing Equations
Governing equations and the initial and boundary conditions for the transportation of hydrogen-natural gas mixture in an inclined pipeline
$\frac{\partial P}{\partial t} + \frac{\partial Pu}{\partial x} = 0,$
$\frac{\partial Pu}{\partial t} + \frac{\partial (Pu^2 + c^2 P)}{\partial x} + \frac{fPu|u|}{2D} + Pg\sin\theta = 0,$
$P(0,t) = P_0(t), \quad u(0,t) = u_0(t),$
$P(L,t) = P_L(t), \quad u(L,t) = u_L(t),$
$P = c^2\rho$
Taylor Series
Approximation for $c(P)$, $\rho(P)$, $c(\phi)$ and $\rho(\phi)$
$c(P) \simeq [c(P)]_{P=0} + \frac{P}{1!}[c(P)]'_{P=0} + \frac{P^2}{2!}[c(P)]''_{P=0}$
$\rho(P) \simeq [\rho(P)]_{P=0} + \frac{P}{1!}[\rho(P)]'_{P=0} + \frac{P^2}{2!}[\rho(P)]''_{P=0}$
$c(\phi) \simeq [c(\phi)]_{\phi=0} + \frac{\phi}{1!}[c(\phi)]'_{\phi=0} + \frac{\phi^2}{2!}[c(\phi)]''_{\phi=0}$
$\rho(\phi) \simeq [\rho(\phi)]_{\phi=0} + \frac{\phi}{1!}[\rho(\phi)]'_{\phi=0} + \frac{\phi^2}{2!}[\rho(\phi)]''_{\phi=0}$
Zero Gradient Control (ZGC)
Pressure Optimization
$\frac{\partial c(P)}{\partial P} = 0 \quad \frac{\partial c(\phi)}{\partial \phi} = 0$
and
$\frac{\partial \rho(P)}{\partial P} = 0 \quad \frac{\partial \rho(\phi)}{\partial \phi} = 0$
Runge-Kutta Method
Regression Polynomial
Variation of maximum mass ratio
Pressure (bar)
$\Delta\phi_{max} = 0.2937$
$\Delta\phi_{max} = 0.2915$
$\Delta\phi_{max} = 0.2910$
$P_0 = 30, 60, 90$
$\text{Pressure Ratio}_{\phi} = \frac{\text{optimal}\{P\}_{\phi=0}}{\text{optimal}\{P\}_{\phi=1}} \simeq 1.22$
$\text{Pressure Ratio}_{P_0} = \frac{\text{optimal}\{P\}_{P_0=30}}{\text{optimal}\{P\}_{P_0=60}} \simeq 1.011$
ϕ: Mass Ratio
P_0: Initial Pressure
P: Transient Pressure
Hydrogen-natural gas mixture (ϕ=0.5) and θ=0
Isothermal
Pressure (bar)
time (s)
Proposed method (NP=100)
Proposed method (NP=10)
Elaoud et al., 2010
Subani et al., 2015
