# Supplementary material for: Controlling the pressure of hydrogen-natural gas mixture in an inclined pipeline
Source: PLoS One. 2020 Feb 27;15(2):e0228955. doi: 10.1371/journal.pone.0228955 (PMC7046196; doi:10.1371/journal.pone.0228955)
Supplement: S1 Program — (PDF) [file pone.0228955.s002.pdf]

Program 1: To approximate Taylor series for Density and Celerity
Maple Code 1: Taylor series for density and celerity

```
restart:with(plots):
A[1]:=(P0/P)^(1/n[1]):
A[2]:=(P0/P)^(1/n[2]):
B[1]:=phi/rho[h0]: B[2]:=(1-phi)/rho[g0]:
rho1:=(B[1]*A[1]+B[2]*A[2])^(-1):
PP3:=convert(series(rho1, phi, 3), polynom);
dphi1:=diff(PP3, phi);
s2:=solve({dphi1=0}, {phi});
eq1:=subs({(P0/P)^(1/n[1])=L[1], (P0/P)^(1/n[2])=L[2]}, PP3);
eq2:=diff(eq1, phi);
eq3:=solve(eq2=0, phi);

restart:with(plots):
A[1]:=(P0/P)^(1/n[1]):
A[2]:=(P0/P)^(1/n[2]):
B[1]:=phi/rho[h0]: B[2]:=(1-phi)/rho[g0]:
rho1:=(B[1]*A[1]+B[2]*A[2])^(-1):
c1:=diff(rho1, P):
c2:=c1^(-1/2):
PP3:=convert(series(c2, phi, 3), polynom);
dphi1:=diff(PP3, phi);
s2:=solve({dphi1=0}, {phi});
```
