# Supplementary material for: Controlling the pressure of hydrogen-natural gas mixture in an inclined pipeline
Source: PLoS One. 2020 Feb 27;15(2):e0228955. doi: 10.1371/journal.pone.0228955 (PMC7046196; doi:10.1371/journal.pone.0228955)
Supplement: S2 Program — (PDF) [file pone.0228955.s003.pdf]

Program 2: To find the regression polynomials for pressure and velocity
Maple Code 2: Regression polynomials for pressure and velocity

```
restart:with(plots):with(Statistics):
Cvh:=10440: Cvg:=1056.8: Cv=phi*Cvh+(1-phi)*Cvg:
Tg=15: Z:=0.995:
Cph:=14600: Cvh:=10440:  Cpg:=1497.5:  Cvg:=1056.8:
# phi:=0: F:=0.007:   ##   phi:=0:
# phi:=0.5: F:=0.18:   ##   phi:=0.5:
# phi:=1: F:=0.61:   ##   phi:=1:
X:=600:  dx:=1:
n:=1.3985: nn:=1.4170:
rho[h0]:=0.00002921*10^0: rho[g0]:=0.0002759*10^0:
d:=0.4: g:=9.8: theta:=0:
Q0:=55: Q0:=18: A:=(Pi/4)*(d^2);
P0:=35: u0=Q1/A:
c:=evalf((phi*exp(ln(P0)/n)/rho[h0]+(1-
phi)*exp(ln(P0)/nn)/rho[g0])/sqrt((phi*exp(ln(P0)/n)*rho[g0]*nn-
exp(ln(P0)/nn)*rho[h0]*n*phi+exp(ln(P0)/nn)*rho[h0]*n)/(rho[h0]*n*rho[g0]*nn))+((phi*exp(l
n(P0)/n)/rho[h0]+(1-phi)*exp(ln(P0)/nn)/rho[g0])*(phi*exp(ln(P0)/n)*n*rho[g0]*nn^2-
exp(ln(P0)/nn)*rho[h0]*n^2*nn*phi+phi*exp(ln(P0)/n)*rho[g0]*nn^2+exp(ln(P0)/nn)*rho[h0]*
n^2*nn-
exp(ln(P0)/nn)*rho[h0]*n^2*phi+exp(ln(P0)/nn)*rho[h0]*n^2)/(2*sqrt((phi*exp(ln(P0)/n)*rho[
g0]*nn-
exp(ln(P0)/nn)*rho[h0]*n*phi+exp(ln(P0)/nn)*rho[h0]*n)/(rho[h0]*n*rho[g0]*nn))*n*nn*(phi
*exp(ln(P0)/n)*rho[g0]*nn-exp(ln(P0)/nn)*rho[h0]*n*phi+exp(ln(P0)/nn)*rho[h0]*n))+(-
phi*exp(ln(P0)/n)/(rho[h0]*n)-(1-
phi)*exp(ln(P0)/nn)/(rho[g0]*nn))/sqrt((phi*exp(ln(P0)/n)*rho[g0]*nn-
exp(ln(P0)/nn)*rho[h0]*n*phi+exp(ln(P0)/nn)*rho[h0]*n)/(rho[h0]*n*rho[g0]*nn)))*ln(P(x))+((
phi*exp(ln(P0)/n)/rho[h0]+(1-phi)*exp(ln(P0)/nn)/rho[g0])*(-
(phi*exp(ln(P0)/n)*n^2*rho[g0]*nn^3-
exp(ln(P0)/nn)*rho[h0]*n^3*nn^2*phi+2*phi*exp(ln(P0)/n)*n*rho[g0]*nn^3+exp(ln(P0)/nn)*r
ho[h0]*n^3*nn^2-
2*exp(ln(P0)/nn)*rho[h0]*n^3*nn*phi+phi*exp(ln(P0)/n)*rho[g0]*nn^3+2*exp(ln(P0)/nn)*rho
[h0]*n^3*nn-
exp(ln(P0)/nn)*rho[h0]*n^3*phi+exp(ln(P0)/nn)*rho[h0]*n^3)/(4*n^2*nn^2*(phi*exp(ln(P0)/n
)*rho[g0]*nn-
exp(ln(P0)/nn)*rho[h0]*n*phi+exp(ln(P0)/nn)*rho[h0]*n))+3*(phi*exp(ln(P0)/n)*n*rho[g0]*nn
^2-
exp(ln(P0)/nn)*rho[h0]*n^2*nn*phi+phi*exp(ln(P0)/n)*rho[g0]*nn^2+exp(ln(P0)/nn)*rho[h0]*
n^2*nn-
exp(ln(P0)/nn)*rho[h0]*n^2*phi+exp(ln(P0)/nn)*rho[h0]*n^2)^2/(8*n^2*nn^2*(phi*exp(ln(P0
)/n)*rho[g0]*nn-
exp(ln(P0)/nn)*rho[h0]*n*phi+exp(ln(P0)/nn)*rho[h0]*n)^2))/sqrt((phi*exp(ln(P0)/n)*rho[g0]*
```

nn-exp(ln(P0)/nn)*rho[h0]*n*phi+exp(ln(P0)/nn)*rho[h0]*n)/(rho[h0]*n*rho[g0]*nn))+(-
phi*exp(ln(P0)/n)/(rho[h0]*n)-(1-
phi)*exp(ln(P0)/nn)/(rho[g0]*nn))*(phi*exp(ln(P0)/n)*n*rho[g0]*nn^2-
exp(ln(P0)/nn)*rho[h0]*n^2*nn*phi+phi*exp(ln(P0)/n)*rho[g0]*nn^2+exp(ln(P0)/nn)*rho[h0]*
n^2*nn-
exp(ln(P0)/nn)*rho[h0]*n^2*phi+exp(ln(P0)/nn)*rho[h0]*n^2)/(2*sqrt((phi*exp(ln(P0)/n)*rho[
g0]*nn-
exp(ln(P0)/nn)*rho[h0]*n*phi+exp(ln(P0)/nn)*rho[h0]*n)/(rho[h0]*n*rho[g0]*nn))*n*nn*(phi
*exp(ln(P0)/n)*rho[g0]*nn-
exp(ln(P0)/nn)*rho[h0]*n*phi+exp(ln(P0)/nn)*rho[h0]*n))+(phi*exp(ln(P0)/n)/(2*rho[h0]*n^2)
+(1-phi)*exp(ln(P0)/nn)/(2*rho[g0]*nn^2))/sqrt((phi*exp(ln(P0)/n)*rho[g0]*nn-
exp(ln(P0)/nn)*rho[h0]*n*phi+exp(ln(P0)/nn)*rho[h0]*n)/(rho[h0]*n*rho[g0]*nn)))*ln(P(x))^2
+((phi*exp(ln(P0)/n)/rho[h0]+(1-
phi)*exp(ln(P0)/nn)/rho[g0])*((phi*exp(ln(P0)/n)*n^3*nn^4*rho[g0]-
exp(ln(P0)/nn)*n^4*rho[h0]*nn^3*phi+3*phi*exp(ln(P0)/n)*n^2*nn^4*rho[g0]+exp(ln(P0)/nn)
*n^4*rho[h0]*nn^3-
3*exp(ln(P0)/nn)*n^4*rho[h0]*nn^2*phi+3*phi*exp(ln(P0)/n)*n*nn^4*rho[g0]+3*exp(ln(P0)/
nn)*n^4*rho[h0]*nn^2-
3*exp(ln(P0)/nn)*n^4*rho[h0]*nn*phi+phi*exp(ln(P0)/n)*nn^4*rho[g0]+3*exp(ln(P0)/nn)*n^4
*rho[h0]*nn-
exp(ln(P0)/nn)*n^4*rho[h0]*phi+exp(ln(P0)/nn)*n^4*rho[h0])/(12*n^3*nn^3*(phi*exp(ln(P0)/
n)*rho[g0]*nn-exp(ln(P0)/nn)*rho[h0]*n*phi+exp(ln(P0)/nn)*rho[h0]*n))-
(3*(phi*exp(ln(P0)/n)*n*rho[g0]*nn^2-
exp(ln(P0)/nn)*rho[h0]*n^2*nn*phi+phi*exp(ln(P0)/n)*rho[g0]*nn^2+exp(ln(P0)/nn)*rho[h0]*
n^2*nn-
exp(ln(P0)/nn)*rho[h0]*n^2*phi+exp(ln(P0)/nn)*rho[h0]*n^2))*(phi*exp(ln(P0)/n)*n^2*rho[g0
]*nn^3-
exp(ln(P0)/nn)*rho[h0]*n^3*nn^2*phi+2*phi*exp(ln(P0)/n)*n*rho[g0]*nn^3+exp(ln(P0)/nn)*r
ho[h0]*n^3*nn^2-
2*exp(ln(P0)/nn)*rho[h0]*n^3*nn*phi+phi*exp(ln(P0)/n)*rho[g0]*nn^3+2*exp(ln(P0)/nn)*rho
[h0]*n^3*nn-
exp(ln(P0)/nn)*rho[h0]*n^3*phi+exp(ln(P0)/nn)*rho[h0]*n^3)/(8*n^3*nn^3*(phi*exp(ln(P0)/n
)*rho[g0]*nn-
exp(ln(P0)/nn)*rho[h0]*n*phi+exp(ln(P0)/nn)*rho[h0]*n)^2)+5*(phi*exp(ln(P0)/n)*n*rho[g0]*
nn^2-
exp(ln(P0)/nn)*rho[h0]*n^2*nn*phi+phi*exp(ln(P0)/n)*rho[g0]*nn^2+exp(ln(P0)/nn)*rho[h0]*
n^2*nn-
exp(ln(P0)/nn)*rho[h0]*n^2*phi+exp(ln(P0)/nn)*rho[h0]*n^2)^3/(16*n^3*nn^3*(phi*exp(ln(P
0)/n)*rho[g0]*nn-
exp(ln(P0)/nn)*rho[h0]*n*phi+exp(ln(P0)/nn)*rho[h0]*n)^3))/sqrt((phi*exp(ln(P0)/n)*rho[g0]*
nn-exp(ln(P0)/nn)*rho[h0]*n*phi+exp(ln(P0)/nn)*rho[h0]*n)/(rho[h0]*n*rho[g0]*nn))+(-
phi*exp(ln(P0)/n)/(rho[h0]*n)-(1-phi)*exp(ln(P0)/nn)/(rho[g0]*nn))*(-
(phi*exp(ln(P0)/n)*n^2*rho[g0]*nn^3-
exp(ln(P0)/nn)*rho[h0]*n^3*nn^2*phi+2*phi*exp(ln(P0)/n)*n*rho[g0]*nn^3+exp(ln(P0)/nn)*r

```
ho[h0]*n^3*nn^2-
2*exp(ln(P0)/nn)*rho[h0]*n^3*nn*phi+phi*exp(ln(P0)/n)*rho[g0]*nn^3+2*exp(ln(P0)/nn)*rho
[h0]*n^3*nn-
exp(ln(P0)/nn)*rho[h0]*n^3*phi+exp(ln(P0)/nn)*rho[h0]*n^3)/(4*n^2*nn^2*(phi*exp(ln(P0)/n
)*rho[g0]*nn-
exp(ln(P0)/nn)*rho[h0]*n*phi+exp(ln(P0)/nn)*rho[h0]*n))+3*(phi*exp(ln(P0)/n)*n*rho[g0]*nn
^2-
exp(ln(P0)/nn)*rho[h0]*n^2*nn*phi+phi*exp(ln(P0)/n)*rho[g0]*nn^2+exp(ln(P0)/nn)*rho[h0]*
n^2*nn-
exp(ln(P0)/nn)*rho[h0]*n^2*phi+exp(ln(P0)/nn)*rho[h0]*n^2)^2/(8*n^2*nn^2*(phi*exp(ln(P0
)/n)*rho[g0]*nn-
exp(ln(P0)/nn)*rho[h0]*n*phi+exp(ln(P0)/nn)*rho[h0]*n)^2))/sqrt((phi*exp(ln(P0)/n)*rho[g0]*
nn-
exp(ln(P0)/nn)*rho[h0]*n*phi+exp(ln(P0)/nn)*rho[h0]*n)/(rho[h0]*n*rho[g0]*nn))+(phi*exp(ln
(P0)/n)/(2*rho[h0]*n^2)+(1-
phi)*exp(ln(P0)/nn)/(2*rho[g0]*nn^2))*(phi*exp(ln(P0)/n)*n*rho[g0]*nn^2-
exp(ln(P0)/nn)*rho[h0]*n^2*nn*phi+phi*exp(ln(P0)/n)*rho[g0]*nn^2+exp(ln(P0)/nn)*rho[h0]*
n^2*nn-
exp(ln(P0)/nn)*rho[h0]*n^2*phi+exp(ln(P0)/nn)*rho[h0]*n^2)/(2*sqrt((phi*exp(ln(P0)/n)*rho[
g0]*nn-
exp(ln(P0)/nn)*rho[h0]*n*phi+exp(ln(P0)/nn)*rho[h0]*n)/(rho[h0]*n*rho[g0]*nn))*n*nn*(phi
*exp(ln(P0)/n)*rho[g0]*nn-exp(ln(P0)/nn)*rho[h0]*n*phi+exp(ln(P0)/nn)*rho[h0]*n))+(-
phi*exp(ln(P0)/n)/(6*rho[h0]*n^3)-(1-
phi)*exp(ln(P0)/nn)/(6*rho[g0]*nn^3))/sqrt((phi*exp(ln(P0)/n)*rho[g0]*nn-
exp(ln(P0)/nn)*rho[h0]*n*phi+exp(ln(P0)/nn)*rho[h0]*n)/(rho[h0]*n*rho[g0]*nn)))*ln(P(x))^3)
;
eq[1]:=1/c^2*1/A^2*diff(P(x)*Q(x), x)=0;
eq[2]:=diff(1*P(x)*Q(x)^2+A^0*c^2*P(x), x)+F*P(x)*Q(x)^2/(2*d)=0;
ini:=P(0)=35, Q(0)=Q0;   ### phi=0.5:
#ini:=P(0)=37, Q(0)=Q0;    ###  phi:=0:
sol:=dsolve({eq[1], eq[2], ini}, numeric);
sol(X);
odeplot(sol,[x,P(x)],0..X, color=blue):
odeplot(sol,[x,Q(x)],0..X, color=blue):
h:=dx:
TT2:=[seq(tt, tt=0..X, h)]:
PP1:=[seq(abs(rhs(sol(x)[2])), x=0..X, h)]:
VV1:=[seq(abs(rhs(sol(x)[3])), x=0..X, h)]:
P3:=Mean(PP1);
u3:=Mean(VV1);
P2:=Fit(a1+a2*x+a3*x^2,TT2,PP1,x);
u2:=Fit(a1+a2*x+a3*x^2,TT2,VV1,x);
plot(P2, x=0..X);
plot(u2, x=0..X);
```
