# Supplementary material for: Controlling the pressure of hydrogen-natural gas mixture in an inclined pipeline
Source: PLoS One. 2020 Feb 27;15(2):e0228955. doi: 10.1371/journal.pone.0228955 (PMC7046196; doi:10.1371/journal.pone.0228955)
Supplement: S3 Program — (PDF) [file pone.0228955.s004.pdf]

Program 3:
Maple Code 3: Solution of equations (2)-(3)

```
restart:with(plots):with(Statistics):with(LinearAlgebra):with(ArrayTools):
phi:=0.25:  X:=600:  Tn:=20:  dx:=5:  a:=3800:  dt=dx/a: dt:=0.61: B:=-2.225:  X:=X/3:
F:=0.03: d:=0.4: g:=9.8: theta:=0:
U:=20: Cv=phi*Cvh+(1-phi)*Cvg:
Tg:=15: Z:=0.995: R:=8.31446*10^(-5):
Cph:=14600: Cvh:=10440:  Cpg:=1497.5:  Cvg:=1056.8:
n:=1.398: nn:=1.4170: rho[h0]:=0.0899: rho[g0]:=0.7171:
Q0:=55: Q0=18: AA1:=(Pi/4*d^2);
P0:=35: u0:=Q0/AA1; T0:=15:
Cp:=phi*Cph+(1-phi)*Cpg:   Cv:=phi*Cvh+(1-phi)*Cvg:
R:=Cp-Cv:
rs1:=0.5:
nomh:=2*rs1: nomg:=12*(1-rs1):
rs:=nomh/(nomh+nomg);
Cvhg:=rs*Cvh+(1-rs)*Cvg;
qx:=4*U/d*(Tg-TT(x));
c:=evalf((phi*exp(ln(P0)/n)/rho[h0]+(1-
phi)*exp(ln(P0)/nn)/rho[g0])/sqrt((phi*exp(ln(P0)/n)*rho[g0]*nn-
exp(ln(P0)/nn)*rho[h0]*n*phi+exp(ln(P0)/nn)*rho[h0]*n)/(rho[h0]*n*rho[g0]*nn))+((phi*exp(l
n(P0)/n)/rho[h0]+(1-phi)*exp(ln(P0)/nn)/rho[g0])*(phi*exp(ln(P0)/n)*n*rho[g0]*nn^2-
exp(ln(P0)/nn)*rho[h0]*n^2*nn*phi+phi*exp(ln(P0)/n)*rho[g0]*nn^2+exp(ln(P0)/nn)*rho[h0]*
n^2*nn-
exp(ln(P0)/nn)*rho[h0]*n^2*phi+exp(ln(P0)/nn)*rho[h0]*n^2)/(2*sqrt((phi*exp(ln(P0)/n)*rho[
g0]*nn-
exp(ln(P0)/nn)*rho[h0]*n*phi+exp(ln(P0)/nn)*rho[h0]*n)/(rho[h0]*n*rho[g0]*nn))*n*nn*(phi
*exp(ln(P0)/n)*rho[g0]*nn-exp(ln(P0)/nn)*rho[h0]*n*phi+exp(ln(P0)/nn)*rho[h0]*n))+(-
phi*exp(ln(P0)/n)/(rho[h0]*n)-(1-
phi)*exp(ln(P0)/nn)/(rho[g0]*nn))/sqrt((phi*exp(ln(P0)/n)*rho[g0]*nn-
exp(ln(P0)/nn)*rho[h0]*n*phi+exp(ln(P0)/nn)*rho[h0]*n)/(rho[h0]*n*rho[g0]*nn)))*ln(P(x))+((
phi*exp(ln(P0)/n)/rho[h0]+(1-phi)*exp(ln(P0)/nn)/rho[g0])*(-
(phi*exp(ln(P0)/n)*n^2*rho[g0]*nn^3-
exp(ln(P0)/nn)*rho[h0]*n^3*nn^2*phi+2*phi*exp(ln(P0)/n)*n*rho[g0]*nn^3+exp(ln(P0)/nn)*r
ho[h0]*n^3*nn^2-
2*exp(ln(P0)/nn)*rho[h0]*n^3*nn*phi+phi*exp(ln(P0)/n)*rho[g0]*nn^3+2*exp(ln(P0)/nn)*rho
[h0]*n^3*nn-
exp(ln(P0)/nn)*rho[h0]*n^3*phi+exp(ln(P0)/nn)*rho[h0]*n^3)/(4*n^2*nn^2*(phi*exp(ln(P0)/n
)*rho[g0]*nn-
exp(ln(P0)/nn)*rho[h0]*n*phi+exp(ln(P0)/nn)*rho[h0]*n))+3*(phi*exp(ln(P0)/n)*n*rho[g0]*nn
^2-
exp(ln(P0)/nn)*rho[h0]*n^2*nn*phi+phi*exp(ln(P0)/n)*rho[g0]*nn^2+exp(ln(P0)/nn)*rho[h0]*
n^2*nn-
```

exp(ln(P0)/nn)*rho[h0]*n^2*phi+exp(ln(P0)/nn)*rho[h0]*n^2)^2/(8*n^2*nn^2*(phi*exp(ln(P0
)/n)*rho[g0]*nn-
exp(ln(P0)/nn)*rho[h0]*n*phi+exp(ln(P0)/nn)*rho[h0]*n)^2))/sqrt((phi*exp(ln(P0)/n)*rho[g0]*
nn-exp(ln(P0)/nn)*rho[h0]*n*phi+exp(ln(P0)/nn)*rho[h0]*n)/(rho[h0]*n*rho[g0]*nn))+(-
phi*exp(ln(P0)/n)/(rho[h0]*n)-(1-
phi)*exp(ln(P0)/nn)/(rho[g0]*nn))*(phi*exp(ln(P0)/n)*n*rho[g0]*nn^2-
exp(ln(P0)/nn)*rho[h0]*n^2*nn*phi+phi*exp(ln(P0)/n)*rho[g0]*nn^2+exp(ln(P0)/nn)*rho[h0]*
n^2*nn-
exp(ln(P0)/nn)*rho[h0]*n^2*phi+exp(ln(P0)/nn)*rho[h0]*n^2)/(2*sqrt((phi*exp(ln(P0)/n)*rho[
g0]*nn-
exp(ln(P0)/nn)*rho[h0]*n*phi+exp(ln(P0)/nn)*rho[h0]*n)/(rho[h0]*n*rho[g0]*nn))*n*nn*(phi
*exp(ln(P0)/n)*rho[g0]*nn-
exp(ln(P0)/nn)*rho[h0]*n*phi+exp(ln(P0)/nn)*rho[h0]*n))+(phi*exp(ln(P0)/n)/(2*rho[h0]*n^2)
+(1-phi)*exp(ln(P0)/nn)/(2*rho[g0]*nn^2))/sqrt((phi*exp(ln(P0)/n)*rho[g0]*nn-
exp(ln(P0)/nn)*rho[h0]*n*phi+exp(ln(P0)/nn)*rho[h0]*n)/(rho[h0]*n*rho[g0]*nn)))*ln(P(x))^2
+((phi*exp(ln(P0)/n)/rho[h0]+(1-
phi)*exp(ln(P0)/nn)/rho[g0])*((phi*exp(ln(P0)/n)*n^3*nn^4*rho[g0]-
exp(ln(P0)/nn)*n^4*rho[h0]*nn^3*phi+3*phi*exp(ln(P0)/n)*n^2*nn^4*rho[g0]+exp(ln(P0)/nn)
*n^4*rho[h0]*nn^3-
3*exp(ln(P0)/nn)*n^4*rho[h0]*nn^2*phi+3*phi*exp(ln(P0)/n)*n*nn^4*rho[g0]+3*exp(ln(P0)/
nn)*n^4*rho[h0]*nn^2-
3*exp(ln(P0)/nn)*n^4*rho[h0]*nn*phi+phi*exp(ln(P0)/n)*nn^4*rho[g0]+3*exp(ln(P0)/nn)*n^4
*rho[h0]*nn-
exp(ln(P0)/nn)*n^4*rho[h0]*phi+exp(ln(P0)/nn)*n^4*rho[h0])/(12*n^3*nn^3*(phi*exp(ln(P0)/
n)*rho[g0]*nn-exp(ln(P0)/nn)*rho[h0]*n*phi+exp(ln(P0)/nn)*rho[h0]*n))-
(3*(phi*exp(ln(P0)/n)*n*rho[g0]*nn^2-
exp(ln(P0)/nn)*rho[h0]*n^2*nn*phi+phi*exp(ln(P0)/n)*rho[g0]*nn^2+exp(ln(P0)/nn)*rho[h0]*
n^2*nn-
exp(ln(P0)/nn)*rho[h0]*n^2*phi+exp(ln(P0)/nn)*rho[h0]*n^2))*(phi*exp(ln(P0)/n)*n^2*rho[g0
]*nn^3-
exp(ln(P0)/nn)*rho[h0]*n^3*nn^2*phi+2*phi*exp(ln(P0)/n)*n*rho[g0]*nn^3+exp(ln(P0)/nn)*r
ho[h0]*n^3*nn^2-
2*exp(ln(P0)/nn)*rho[h0]*n^3*nn*phi+phi*exp(ln(P0)/n)*rho[g0]*nn^3+2*exp(ln(P0)/nn)*rho
[h0]*n^3*nn-
exp(ln(P0)/nn)*rho[h0]*n^3*phi+exp(ln(P0)/nn)*rho[h0]*n^3)/(8*n^3*nn^3*(phi*exp(ln(P0)/n
)*rho[g0]*nn-
exp(ln(P0)/nn)*rho[h0]*n*phi+exp(ln(P0)/nn)*rho[h0]*n)^2)+5*(phi*exp(ln(P0)/n)*n*rho[g0]*
nn^2-
exp(ln(P0)/nn)*rho[h0]*n^2*nn*phi+phi*exp(ln(P0)/n)*rho[g0]*nn^2+exp(ln(P0)/nn)*rho[h0]*
n^2*nn-
exp(ln(P0)/nn)*rho[h0]*n^2*phi+exp(ln(P0)/nn)*rho[h0]*n^2)^3/(16*n^3*nn^3*(phi*exp(ln(P
0)/n)*rho[g0]*nn-
exp(ln(P0)/nn)*rho[h0]*n*phi+exp(ln(P0)/nn)*rho[h0]*n)^3))/sqrt((phi*exp(ln(P0)/n)*rho[g0]*
nn-exp(ln(P0)/nn)*rho[h0]*n*phi+exp(ln(P0)/nn)*rho[h0]*n)/(rho[h0]*n*rho[g0]*nn))+(-

```
phi*exp(ln(P0)/n)/(rho[h0]*n)-(1-phi)*exp(ln(P0)/nn)/(rho[g0]*nn))*(-
(phi*exp(ln(P0)/n)*n^2*rho[g0]*nn^3-
exp(ln(P0)/nn)*rho[h0]*n^3*nn^2*phi+2*phi*exp(ln(P0)/n)*n*rho[g0]*nn^3+exp(ln(P0)/nn)*r
ho[h0]*n^3*nn^2-
2*exp(ln(P0)/nn)*rho[h0]*n^3*nn*phi+phi*exp(ln(P0)/n)*rho[g0]*nn^3+2*exp(ln(P0)/nn)*rho
[h0]*n^3*nn-
exp(ln(P0)/nn)*rho[h0]*n^3*phi+exp(ln(P0)/nn)*rho[h0]*n^3)/(4*n^2*nn^2*(phi*exp(ln(P0)/n
)*rho[g0]*nn-
exp(ln(P0)/nn)*rho[h0]*n*phi+exp(ln(P0)/nn)*rho[h0]*n))+3*(phi*exp(ln(P0)/n)*n*rho[g0]*nn
^2-
exp(ln(P0)/nn)*rho[h0]*n^2*nn*phi+phi*exp(ln(P0)/n)*rho[g0]*nn^2+exp(ln(P0)/nn)*rho[h0]*
n^2*nn-
exp(ln(P0)/nn)*rho[h0]*n^2*phi+exp(ln(P0)/nn)*rho[h0]*n^2)^2/(8*n^2*nn^2*(phi*exp(ln(P0
)/n)*rho[g0]*nn-
exp(ln(P0)/nn)*rho[h0]*n*phi+exp(ln(P0)/nn)*rho[h0]*n)^2))/sqrt((phi*exp(ln(P0)/n)*rho[g0]*
nn-
exp(ln(P0)/nn)*rho[h0]*n*phi+exp(ln(P0)/nn)*rho[h0]*n)/(rho[h0]*n*rho[g0]*nn))+(phi*exp(ln
(P0)/n)/(2*rho[h0]*n^2)+(1-
phi)*exp(ln(P0)/nn)/(2*rho[g0]*nn^2))*(phi*exp(ln(P0)/n)*n*rho[g0]*nn^2-
exp(ln(P0)/nn)*rho[h0]*n^2*nn*phi+phi*exp(ln(P0)/n)*rho[g0]*nn^2+exp(ln(P0)/nn)*rho[h0]*
n^2*nn-
exp(ln(P0)/nn)*rho[h0]*n^2*phi+exp(ln(P0)/nn)*rho[h0]*n^2)/(2*sqrt((phi*exp(ln(P0)/n)*rho[
g0]*nn-
exp(ln(P0)/nn)*rho[h0]*n*phi+exp(ln(P0)/nn)*rho[h0]*n)/(rho[h0]*n*rho[g0]*nn))*n*nn*(phi
*exp(ln(P0)/n)*rho[g0]*nn-exp(ln(P0)/nn)*rho[h0]*n*phi+exp(ln(P0)/nn)*rho[h0]*n))+(-
phi*exp(ln(P0)/n)/(6*rho[h0]*n^3)-(1-
phi)*exp(ln(P0)/nn)/(6*rho[g0]*nn^3))/sqrt((phi*exp(ln(P0)/n)*rho[g0]*nn-
exp(ln(P0)/nn)*rho[h0]*n*phi+exp(ln(P0)/nn)*rho[h0]*n)/(rho[h0]*n*rho[g0]*nn)))*ln(P(x))^3)
;
c:=c/10^B;
g1:=9.8:
ee1:=Cvhg*TT(x):
eq[1]:=diff(P(x)*u(x), x)=0:
eq[2]:=diff(P(x)*u(x)^2, x)+c^2*diff(P(x), x)+F*P(x)*u(x)*abs(u(x))/(2*d)+P(x)*g1*sin(theta)=0:
ini:=P(0)=P0, u(0)=u0;     ### phi=0.5:
sol:=dsolve({eq[1], eq[2], ini}, numeric);
sol(X);
odeplot(sol,[x,P(x)],0..X, color=blue):
odeplot(sol,[x,u(x)],0..X, color=blue):
h:=1:
TT2:=[seq(tt, tt=0..X, h)]:
PP1:=[seq(abs(rhs(sol(x)[2])), x=0..X, h)]:
VV1:=[seq(abs(rhs(sol(x)[3])), x=0..X, h)]:
P2:=Fit(a1+a2*x+a3*x^2,TT2,PP1,x);
```

```
u2:=Fit(a1+a2*x+a3*x^2,TT2,VV1,x);
plot(P2, x=0..X);
plot(u2, x=0..X):
cc=a:
cc:=evalf((phi*exp(ln(P0)/n)/rho[h0]+(1-
phi)*exp(ln(P0)/nn)/rho[g0])/sqrt((phi*exp(ln(P0)/n)*rho[g0]*nn-
exp(ln(P0)/nn)*rho[h0]*n*phi+exp(ln(P0)/nn)*rho[h0]*n)/(rho[h0]*n*rho[g0]*nn))+((phi*exp(l
n(P0)/n)/rho[h0]+(1-phi)*exp(ln(P0)/nn)/rho[g0])*(phi*exp(ln(P0)/n)*n*rho[g0]*nn^2-
exp(ln(P0)/nn)*rho[h0]*n^2*nn*phi+phi*exp(ln(P0)/n)*rho[g0]*nn^2+exp(ln(P0)/nn)*rho[h0]*
n^2*nn-
exp(ln(P0)/nn)*rho[h0]*n^2*phi+exp(ln(P0)/nn)*rho[h0]*n^2)/(2*sqrt((phi*exp(ln(P0)/n)*rho[
g0]*nn-
exp(ln(P0)/nn)*rho[h0]*n*phi+exp(ln(P0)/nn)*rho[h0]*n)/(rho[h0]*n*rho[g0]*nn))*n*nn*(phi
*exp(ln(P0)/n)*rho[g0]*nn-exp(ln(P0)/nn)*rho[h0]*n*phi+exp(ln(P0)/nn)*rho[h0]*n))+(-
phi*exp(ln(P0)/n)/(rho[h0]*n)-(1-
phi)*exp(ln(P0)/nn)/(rho[g0]*nn))/sqrt((phi*exp(ln(P0)/n)*rho[g0]*nn-
exp(ln(P0)/nn)*rho[h0]*n*phi+exp(ln(P0)/nn)*rho[h0]*n)/(rho[h0]*n*rho[g0]*nn)))*ln(P1(x,
t))+((phi*exp(ln(P0)/n)/rho[h0]+(1-phi)*exp(ln(P0)/nn)/rho[g0])*(-
(phi*exp(ln(P0)/n)*n^2*nn^3*rho[g0]-
exp(ln(P0)/nn)*n^3*rho[h0]*nn^2*phi+2*phi*exp(ln(P0)/n)*n*nn^3*rho[g0]+exp(ln(P0)/nn)*n
^3*rho[h0]*nn^2-
2*exp(ln(P0)/nn)*n^3*rho[h0]*nn*phi+phi*exp(ln(P0)/n)*nn^3*rho[g0]+2*exp(ln(P0)/nn)*n^3
*rho[h0]*nn-
exp(ln(P0)/nn)*n^3*rho[h0]*phi+exp(ln(P0)/nn)*n^3*rho[h0])/(4*n^2*nn^2*(phi*exp(ln(P0)/n
)*rho[g0]*nn-
exp(ln(P0)/nn)*rho[h0]*n*phi+exp(ln(P0)/nn)*rho[h0]*n))+3*(phi*exp(ln(P0)/n)*n*rho[g0]*nn
^2-
exp(ln(P0)/nn)*rho[h0]*n^2*nn*phi+phi*exp(ln(P0)/n)*rho[g0]*nn^2+exp(ln(P0)/nn)*rho[h0]*
n^2*nn-
exp(ln(P0)/nn)*rho[h0]*n^2*phi+exp(ln(P0)/nn)*rho[h0]*n^2)^2/(8*n^2*nn^2*(phi*exp(ln(P0
)/n)*rho[g0]*nn-
exp(ln(P0)/nn)*rho[h0]*n*phi+exp(ln(P0)/nn)*rho[h0]*n)^2))/sqrt((phi*exp(ln(P0)/n)*rho[g0]*
nn-exp(ln(P0)/nn)*rho[h0]*n*phi+exp(ln(P0)/nn)*rho[h0]*n)/(rho[h0]*n*rho[g0]*nn))+(-
phi*exp(ln(P0)/n)/(rho[h0]*n)-(1-
phi)*exp(ln(P0)/nn)/(rho[g0]*nn))*(phi*exp(ln(P0)/n)*n*rho[g0]*nn^2-
exp(ln(P0)/nn)*rho[h0]*n^2*nn*phi+phi*exp(ln(P0)/n)*rho[g0]*nn^2+exp(ln(P0)/nn)*rho[h0]*
n^2*nn-
exp(ln(P0)/nn)*rho[h0]*n^2*phi+exp(ln(P0)/nn)*rho[h0]*n^2)/(2*sqrt((phi*exp(ln(P0)/n)*rho[
g0]*nn-
exp(ln(P0)/nn)*rho[h0]*n*phi+exp(ln(P0)/nn)*rho[h0]*n)/(rho[h0]*n*rho[g0]*nn))*n*nn*(phi
*exp(ln(P0)/n)*rho[g0]*nn-
exp(ln(P0)/nn)*rho[h0]*n*phi+exp(ln(P0)/nn)*rho[h0]*n))+(phi*exp(ln(P0)/n)/(2*rho[h0]*n^2)
+(1-phi)*exp(ln(P0)/nn)/(2*rho[g0]*nn^2))/sqrt((phi*exp(ln(P0)/n)*rho[g0]*nn-
```

```
exp(ln(P0)/nn)*rho[h0]*n*phi+exp(ln(P0)/nn)*rho[h0]*n)/(rho[h0]*n*rho[g0]*nn)))*ln(P1(x,
t))^2):
cc:=cc/10^B:
cc=1050:
ee2:=Cvhg*TT1(x,t):
qx:=4*U/d*(Tg-TT1(x, t)):
eq[1]:=diff(P1(x, t), t)+diff(P1(x, t)*u1(x, t), x)=0:
eq[2]:=diff(P1(x, t)*u1(x, t), t)+diff(g1*P1(x, t)*u1(x,t)^2+cc^2*P1(x,t), x)+F*P1(x, t)*u1(x,
t)*abs(u1(x, t))/(2*d)+P1(x, t)*g1*sin(theta)=0:
Tl:=0.6; L:=2.2:
ini:= P1(X, t)=piecewise(t<Tl, P0  , t>Tl and t<2*Tl, 48/100*P0+L, t>2*Tl, 73/100*P0+L), u1(X,
t)=u0, P1(x, 0)=P2, u1(x, 0)=u2:
PDE:={eq[1], eq[2]}:
#ss:=pdsolve(PDE,{ini},numeric, time=t, range=0..X,  timestep=dt);
ss:=pdsolve(PDE,{ini},numeric, time=t, range=0..X,  timestep=dt);
#ss:=pdsolve(PDE,{ini},numeric, time=t, range=0..X);
#ss[plot](t=Tn/3, color=red, numpoints=500);
K:=200: X1:=0:
p1[1]:=plots[display](ss:-plot(P1, x=X1, t=0.. Tn, numpoints=K, labels = ["time (s)", "Pressure
(bar)"], labeldirections=[horizontal,vertical])):
#plots[display](ss:-plot(u1, x=X1, t=0.. Tn, numpoints=K, labels = ["time (s)", "Velocity (m/s)"],
labeldirections=[horizontal,vertical]));
#plots[display](ss:-plot((TT1), x=X1, t=0.. Tn, numpoints=K, labels = ["time (s)", "Temperature
(°C)"], labeldirections=[horizontal,vertical]));
#ss:-plot3d(P1,t=0..20,x=0..X,axes=boxed,color=green, numpoints=5000);
###  Phi=0.25, Theta=0,    Ishotherma
S1:=<<0.0558742|34.9654>,<0.821272|27.3803>,<1.00141|28.7213>,<1.1987|29.346>,<1.495
28|29.5667>,<1.64286|30.4484>,<1.87292|31.2936>,<2.03716|31.9917>,<2.15182|32.8183>,
<2.41501|33.4981>,<2.6464|32.8922>,<2.84493|32.1577>,<3.01021|31.7171>,<3.24037|32.4
52>,<3.4707|33.0032>,<3.668|33.6095>,<3.79926|34.3076>,<4.04598|34.969>,<4.22785|34.4
182>,<4.40966|33.9408>,<4.57499|33.445>,<4.82277|32.9493>,<5.03605|34.1067>,<5.21781
|33.6844>,<5.39946|33.3723>,<5.64722|32.895>,<5.8782|32.7299>,<6.04259|33.2627>,<6.22
437|32.822>,<6.4216|33.5018>,<6.70111|34.3653>,<6.84894|34.9716>,<7.09554|35.7616>,<
7.24447|35.174>,<7.47576|34.6783>,<7.62476|34.0172>,<7.83952|33.5582>,<8.12037|32.95
24>,<8.3011|33.6505>,<8.51503|34.1099>,<8.6629|34.6794>,<8.84366|35.3408>,<9.17285|3
5.9656>,<9.32176|35.3963>,<9.5531|34.8455>,<9.71852|34.2579>,<9.88399|33.6152>,<10.18
13|33.0828>,<10.329|33.7442>,<10.4769|34.3504>,<10.6412|34.9751>,<10.9209|35.6549>,<
11.0852|36.2061>,<11.333|35.6737>,<11.548|34.9943>,<11.7463|34.5353>,<11.9118|33.892
6>,<12.0772|33.2682>,<12.3068|34.6827>,<12.4721|34.2053>,<12.6869|33.7096>,<12.9509|
33.4343>,<13.1154|33.9304>,<13.2476|33.5081>,<13.5602|34.2431>,<13.741|34.9412>,<13.9
052|35.676>,<14.1023|36.5027>,<14.3337|35.8417>,<14.598|35.2726>,<14.7636|34.5748>,<
14.9454|34.079>,<15.0614|33.4546>,<15.3246|34.061>,<15.5549|34.6857>,<15.7522|35.310
4>,<15.933|35.8616>,<16.1962|36.5598>,<16.4112|35.862>,<16.5271|35.3478>,<16.7419|34.
797>,<16.9238|34.2094>,<17.0729|33.53>,<17.3525|34.2098>,<17.5169|34.8161>,<17.7636|
```

```
35.4224>,<17.9279|36.0287>,<18.1911|36.6902>,<18.406|36.0843>,<18.5715|35.4783>,<18.7
369|34.9274>,<18.9684|34.1929>,<19.1832|33.7156>,<19.3468|35.0565>,<19.5615|34.6343>
,<19.7437|33.7528>,<20.0069|34.4327>>;
#### Phi=0.5, Theta=0,   Isothermal
S2:=<<0.04971|35.0335>,<0.8285|27.6321>,<1.04391|29.2377>,<1.24275|29.9315>,<1.42502
|30.2422>,<1.60729|31.41>,<1.8227|32.4503>,<1.9884|33.3628>,<2.20381|34.3119>,<2.4192
2|35.188>,<2.63463|34.4046>,<2.80033|33.4751>,<3.03231|32.9836>,<3.24772|33.7686>,<3.
41342|34.5898>,<3.6454|35.4114>,<3.8111|36.1414>,<4.02651|36.9629>,<4.34134|36.344>,
<4.50704|35.6698>,<4.67274|35.1233>,<4.85501|34.5404>,<5.02071|36>,<5.21955|35.5448>
,<5.45153|34.962>,<5.6338|34.525>,<5.83264|34.2704>,<6.06462|34.8731>,<6.23032|34.344
8>,<6.51201|35.2394>,<6.66114|36.0606>,<6.87655|37.0827>,<7.05882|37.9405>,<7.27423|
37.2483>,<7.50621|36.5926>,<7.70505|35.9186>,<7.93703|35.1534>,<8.10273|34.4793>,<8.3
1814|35.2096>,<8.50041|36.0491>,<8.71582|36.7064>,<8.9478|37.528>,<9.09693|38.2397>,
<9.31234|37.511>,<9.56089|36.8554>,<9.71002|36.163>,<9.89229|35.5071>,<10.1408|34.65
08>,<10.3231|35.4722>,<10.4888|36.2935>,<10.7042|37.0237>,<10.9196|37.754>,<11.1516|
38.5026>,<11.251|37.9376>,<11.5327|37.0815>,<11.7481|36.3892>,<11.9138|35.7151>,<12.1
458|34.8952>,<12.3115|36.6284>,<12.5269|36.0273>,<12.8086|35.4083>,<12.9246|35.0987>
,<13.1732|35.7197>,<13.3389|35.1184>,<13.5874|36.0677>,<13.7531|36.9984>,<14.0017|37.
9476>,<14.1674|38.8236>,<14.3331|38.0765>,<14.5319|37.4207>,<14.7473|36.6737>,<14.94
61|35.945>,<15.1947|35.107>,<15.3935|35.9101>,<15.5758|36.7497>,<15.7415|37.498>,<16.
0066|38.2649>,<16.1723|39.0133>,<16.3877|38.2299>,<16.57|37.4646>,<16.7357|36.7539>,
<17.0174|35.9708>,<17.1665|35.2965>,<17.4482|36.0818>,<17.5973|36.83>,<17.7796|37.59
66>,<18.0447|38.3453>,<18.1773|39.203>,<18.3761|38.456>,<18.5915|37.6178>,<18.807|36.
8891>,<19.0058|36.1604>,<19.2543|35.3771>,<19.4366|37.1468>,<19.6023|36.5273>,<19.80
12|35.5615>,<20.0331|36.2371>>:
### Phi=0.5, Theta=0.15,    Isothermal
S3:=<<0|24.2764>,<0.232365|24.8783>,<0.365145|25.5613>,<0.580913|26.0818>,<0.746888|
26.3258>,<1.11203|27.5456>,<1.77593|29.5299>,<2.39004|29.3678>,<2.77178|28.9942>,<3.1
5353|29.531>,<3.55187|30.3606>,<3.83402|30.8324>,<4.61411|30.0851>,<4.82988|30.7682>
,<5.37759|30.1345>,<5.64315|30.0209>,<5.77593|30.2324>,<6.05809|30.0863>,<6.47303|30.
9646>,<6.87137|31.7617>,<7.38589|31.0467>,<7.86722|30.3154>,<8.31535|31.1287>,<8.697
1|31.812>,<8.86307|32.0235>,<9.12863|31.7148>,<9.41079|31.3248>,<9.65975|30.8371>,<9.
85892|30.4796>,<10.2573|31.0978>,<10.5394|31.6671>,<10.8548|32.2365>,<11.1867|31.862
8>,<11.4855|31.3915>,<11.8672|30.6926>,<12.083|31.3757>,<12.5145|30.937>,<12.7303|30.
7909>,<12.9129|31.0024>,<13.0788|30.8074>,<13.4772|31.5069>,<13.9419|32.4179>,<14.27
39|31.9141>,<14.6224|31.3778>,<14.9378|30.9065>,<15.2863|31.5084>,<15.6349|31.9477>,
<15.9336|32.5333>,<16.2324|32.0783>,<16.6141|31.5908>,<16.9959|31.0057>,<17.3776|31.
6239>,<17.5602|32.0793>,<17.9917|32.6976>,<18.2739|32.21>,<18.6224|31.7225>,<19.0041
|31.1049>,<19.1701|31.8205>,<19.5021|31.4956>,<19.7842|31.2194>,<20|31.3984>>:
#### Phi=0.5, Theta=0,   Non Isothermal
S4:=<<0.04971|35.0335>,<0.8285|27.6321>,<1.04391|29.2377>,<1.24275|29.9315>,<1.42502
|30.2422>,<1.60729|31.41>,<1.8227|32.4503>,<1.9884|33.3628>,<2.20381|34.3119>,<2.4192
2|35.188>,<2.63463|34.4046>,<2.80033|33.4751>,<3.03231|32.9836>,<3.24772|33.7686>,<3.
41342|34.5898>,<3.6454|35.4114>,<3.8111|36.1414>,<4.02651|36.9629>,<4.34134|36.344>,
```

```
<4.50704|35.6698>,<4.67274|35.1233>,<4.85501|34.5404>,<5.02071|36>,<5.21955|35.5448>
,<5.45153|34.962>,<5.6338|34.525>,<5.83264|34.2704>,<6.06462|34.8731>,<6.23032|34.344
8>,<6.51201|35.2394>,<6.66114|36.0606>,<6.87655|37.0827>,<7.05882|37.9405>,<7.27423|
37.2483>,<7.50621|36.5926>,<7.70505|35.9186>,<7.93703|35.1534>,<8.10273|34.4793>,<8.3
1814|35.2096>,<8.50041|36.0491>,<8.71582|36.7064>,<8.9478|37.528>,<9.09693|38.2397>,
<9.31234|37.511>,<9.56089|36.8554>,<9.71002|36.163>,<9.89229|35.5071>,<10.1408|34.65
08>,<10.3231|35.4722>,<10.4888|36.2935>,<10.7042|37.0237>,<10.9196|37.754>,<11.1516|
38.5026>,<11.251|37.9376>,<11.5327|37.0815>,<11.7481|36.3892>,<11.9138|35.7151>,<12.1
458|34.8952>,<12.3115|36.6284>,<12.5269|36.0273>,<12.8086|35.4083>,<12.9246|35.0987>
,<13.1732|35.7197>,<13.3389|35.1184>,<13.5874|36.0677>,<13.7531|36.9984>,<14.0017|37.
9476>,<14.1674|38.8236>,<14.3331|38.0765>,<14.5319|37.4207>,<14.7473|36.6737>,<14.94
61|35.945>,<15.1947|35.107>,<15.3935|35.9101>,<15.5758|36.7497>,<15.7415|37.498>,<16.
0066|38.2649>,<16.1723|39.0133>,<16.3877|38.2299>,<16.57|37.4646>,<16.7357|36.7539>,
<17.0174|35.9708>,<17.1665|35.2965>,<17.4482|36.0818>,<17.5973|36.83>,<17.7796|37.59
66>,<18.0447|38.3453>,<18.1773|39.203>,<18.3761|38.456>,<18.5915|37.6178>,<18.807|36.
8891>,<19.0058|36.1604>,<19.2543|35.3771>,<19.4366|37.1468>,<19.6023|36.5273>,<19.80
12|35.5615>,<20.0331|36.2371>>:
#### Phi=0.5, Theta=0.15,   Isothermal
S5:=<<0.03314|35.0791>,<0.81193|23.1839>,<0.9942|25.1959>,<1.22618|25.9909>,<1.47473
|26.4126>,<1.64043|27.6133>,<1.77299|28.6842>,<1.97183|29.8199>,<2.20381|30.8583>,<2.
41922|31.8479>,<2.63463|30.9877>,<2.86661|30.5332>,<3.01574|29.5107>,<3.21458|30.386
8>,<3.46313|31.2954>,<3.6454|32.0579>,<3.91052|32.9826>,<4.07622|33.9399>,<4.25849|3
3.1933>,<4.4739|32.544>,<4.6396|31.8786>,<4.83844|31.1969>,<5.05385|32.933>,<5.26926|
32.3>,<5.4681|31.7643>,<5.70008|31.18>,<5.89892|30.9526>,<6.03148|31.6503>,<6.24689|3
1.0822>,<6.49544|32.088>,<6.69428|33.0453>,<6.92626|34.0349>,<7.09196|35.0084>,<7.290
8|34.1969>,<7.48964|33.5152>,<7.67191|32.7199>,<7.93703|31.9733>,<8.15244|31.2104>,<
8.30157|32.0541>,<8.51698|32.9302>,<8.68268|33.709>,<8.91466|34.5688>,<9.09693|35.36
38>,<9.31234|34.5036>,<9.49461|33.7732>,<9.74316|33.0266>,<9.85915|32.2314>,<10.1243
|31.5009>,<10.3231|32.4257>,<10.5054|33.2045>,<10.7042|34.0481>,<10.9693|34.7781>,<1
1.0853|35.6543>,<11.3505|34.7129>,<11.5327|33.9988>,<11.7316|33.2846>,<11.9138|32.42
44>,<12.1127|31.7265>,<12.4109|33.5761>,<12.5601|32.8782>,<12.792|32.1803>,<12.9412|
31.9368>,<13.1566|32.6668>,<13.3057|31.9851>,<13.5543|32.9586>,<13.7531|34.0619>,<13.
9519|35.0029>,<14.1508|35.9601>,<14.3828|35.1486>,<14.565|34.3371>,<14.7804|33.5093>
,<14.9793|32.7465>,<15.1616|31.9674>,<15.3438|32.8598>,<15.6089|33.7034>,<15.7581|34.
5308>,<15.9569|35.3907>,<16.1723|36.2019>,<16.3546|35.3255>,<16.6031|34.4652>,<16.80
2|33.7024>,<17.034|32.9071>,<17.1831|32.1118>,<17.3819|32.9879>,<17.5642|33.8316>,<1
7.8459|34.6103>,<18.0116|35.5838>,<18.2436|36.3625>,<18.3927|35.4212>,<18.6081|34.59
34>,<18.7904|33.8468>,<19.0224|33.0515>,<19.2543|32.2237>,<19.42|34.1546>,<19.6023|3
3.4567>,<19.8012|32.4991>,<20.0497|33.2129>>:
p1[2]:=plot(S5, color=green):
display(p1[1], p1[2]);
#plot([S2, S5]);
```
