# Supplementary material for: Controlling the pressure of hydrogen-natural gas mixture in an inclined pipeline
Source: PLoS One. 2020 Feb 27;15(2):e0228955. doi: 10.1371/journal.pone.0228955 (PMC7046196; doi:10.1371/journal.pone.0228955)
Supplement: S4 Program — (PDF) [file pone.0228955.s005.pdf]

Program 4:
Maple Code 4: Zero gradient control (ZGC) for pressure control

```
restart:with(plots):
#P0:=35: phi:=0.1: n:=1.3985: nn:=1.4170: rho[h0]:=0.00002921*10^5:
rho[g0]:=0.0002759*10^5:
c1:=phi/rho[h0]*(P0/P)^(1/n)+(1-phi)/rho[g0]*(P0/P)^(1/nn):
c2:=1/P*(1/n*phi/rho[h0]*(P0/P)^(1/n)+1/nn*(1-phi)/rho[g0]*(P0/P)^(1/nn)):
cc:=c1*c2^(-1/2);
cc1:=subs(P=exp(P),cc):
cc2:=convert(taylor(cc1, P, 3), polynom):
p1:=subs(P=ln(P), cc2):
dP:=evalf(diff(p1, P)):
optP:=solve(dP=0, P):
p1:=evalf(subs({P0=35, n=1.3985, nn=1.4170, rho[h0]=0.00002921*10^5,
rho[g0]=0.0002759*10^5}, optP)):
p2:=subs({phi=0.48, n=1.3985, nn=1.4170, rho[h0]=0.00002921*10^5,
rho[g0]=0.0002759*10^5}, optP):
plot(p2, P0=30..60, axes=box, labels = ["Initial pressure", "Optimum pressure"],
labeldirections=[horizontal,vertical]);
plot(p1, phi=0..1, axes=box, labels = ["Mass ratio", "Optimum pressure"],
labeldirections=[horizontal,vertical]);

restart:with(plots):
rho1:=((phi/rho[h0]*(P0/P)^(1/n)+(1-phi)/rho[g0]*(P0/P)^(1/nn)))^(-1);
cc1:=subs(P=exp(P),rho1):
cc2:=convert(taylor(cc1, P, 3), polynom):
p1:=subs(P=ln(P), cc2):
dP:=evalf(diff(p1, P)):
optP:=solve(dP=0, P):
p1:=evalf(subs({P0=35, n=1.3985, nn=1.4170, rho[h0]=0.00002921*10^5,
rho[g0]=0.0002759*10^5}, optP)):
p2:=subs({phi=0.5, n=1.3985, nn=1.4170, rho[h0]=0.00002921*10^5,
rho[g0]=0.0002759*10^5}, optP):
plot(p2, P0=30..60, axes=box, labels = ["Initial pressure", "Optimum pressure"],
labeldirections=[horizontal,vertical]);
plot(p1, phi=0..1, axes=box, labels = ["Mass ratio", "Optimum pressure"],
labeldirections=[horizontal,vertical]);
```
