# Supplementary material for: Controlling the pressure of hydrogen-natural gas mixture in an inclined pipeline
Source: PLoS One. 2020 Feb 27;15(2):e0228955. doi: 10.1371/journal.pone.0228955 (PMC7046196; doi:10.1371/journal.pone.0228955)
Supplement: S5 Program — (PDF) [file pone.0228955.s006.pdf]

Program 5:
Maple Code 5: Pressure control by reining mass ratio

```
restart:with(plots):
# n:=1.398: nn:=1.4170: rho[h0]:=0.0899: rho[g0]:=0.7171:
A[1]:=(P0/P)^(1/n[1]):
A[2]:=(P0/P)^(1/n[2]):
B[1]:=phi/rho[h0]: B[2]:=(1-phi)/rho[g0]:
rho1:=(B[1]*A[1]+B[2]*A[2])^(-1):
P1:=subs({P=exp(P)}, rho1):
PP2:=convert(series(P1, P, 2), polynom):
PP2:=subs(P=ln(P), PP2):
PP3:=convert(series(rho1, phi, 3), polynom):
dP1:=diff(PP2, P):
dphi1:=diff(PP3, phi):
s2:=solve({dphi1=0}, {phi});
P3:=evalf(subs({ P0=90, n[1]=1.398, n[2]=1.4170, rho[h0]=0.0899, rho[g0]=0.7171 }, rhs(s2[1]))):
P4:=evalf(subs({ P=90, n[1]=1.398, n[2]=1.4170, rho[h0]=0.0899, rho[g0]=0.7171 }, rhs(s2[1]))):
P[1]:=plot(P3, P=30..90, color=red, labels = ["Pressure (bar)", "Optimal mass
ratio"],labeldirections=[horizontal,vertical]);
P[2]:=plot(P4, P0=30..90, color=green, axes=box, labels = ["Pressure (bar)", "Optimal mass
ratio"],labeldirections=[horizontal,vertical]);
display(P[1], P[2]);

restart: with(plots):
A[1]:=(P0/P)^(1/n[1]):
A[2]:=(P0/P)^(1/n[2]):
B[1]:=phi/rho[h0]: B[2]:=(1-phi)/rho[g0]:
rho1:=(B[1]*A[1]+B[2]*A[2])^(-1):
c1:=diff(rho1, P):
c2:=c1^(-1/2):
PP1:=convert(series(c2, phi, 3), polynom):
dphi1:=diff(PP1, phi):
s2:=solve({dphi1=0}, {phi}):
P1:=evalf(subs({ P0=90, n[1]=1.398, n[2]=1.4170, rho[h0]=0.0899, rho[g0]=0.7171 }, rhs(s2[1]))):
P4:=evalf(subs({ P=90, n[1]=1.398, n[2]=1.4170, rho[h0]=0.0899, rho[g0]=0.7171 }, rhs(s2[1]))):
P[1]:=plot(P1, P=30..90, color=red, labels = ["Pressure (bar)", "Optimal mass
ratio"],labeldirections=[horizontal,vertical]);
P[2]:=plot(P4, P0=30..90, color=green, axes=box, labels = ["Pressure (bar)", "Mass
ratio"],labeldirections=[horizontal,vertical]);
display(P[1], P[2]);
```
