# Supplementary material for: Controlling the pressure of hydrogen-natural gas mixture in an inclined pipeline
Source: PLoS One. 2020 Feb 27;15(2):e0228955. doi: 10.1371/journal.pone.0228955 (PMC7046196; doi:10.1371/journal.pone.0228955)
Supplement: Attachment — Submitted filename: PONE-D-19-28982_Review.pdf [file pone.0228955.s007.pdf]

PLOS ONE

Controlling optimal pressure of hydrogen-natural gas mixture in an inclined pipeline

--Manuscript Draft--

**Manuscript Number:**	PONE-D-19-28982
**Article Type:**	Research Article
**Full Title:**	Controlling optimal pressure of hydrogen-natural gas mixture in an inclined pipeline
**Short Title:**	Controlling pressure of hydrogen-natural gas mixture in an inclined pipeline
**Corresponding Author:**	Sarkhosh S. Chaharborj<br>Universiti Teknologi Malaysia<br>Johor Bahru, MALAYSIA
**Keywords:**	hydrogen, natural gas, gas mixture, Taylor series, regression analysis, Zero Gradient Control
**Abstract:**	This paper discusses the control of optimal pressure using the zero-gradient control (ZGC) approach. It is applied for the first time in the study to control the optimal pressure of hydrogen natural gas mixture in an inclined pipeline. The solution to the flow problem is first validated with existing results using the Taylor series approximation, regression analysis and the RungeKutta method combined. The optimal pressure is then determined using ZGC where the optimal set points are calculated without having to solve the non-linear system of equations associated with the standard optimization problem. It is shown that the mass ratio is the more effective parameter compared to the initial pressure in controlling the maximum variation of pressure in a gas pipeline
**Order of Authors:**	sarkhosh seddighi chaharborj
	Sarkhosh S. Chaharborj
	Norsarahaida Amin
**Additional Information:**	
**Question**	**Response**
**Financial Disclosure**<br><br>Enter a financial disclosure statement that describes the sources of funding for the work included in this submission. Review the submission guidelines for detailed requirements. View published research articles from *PLOS ONE* for specific examples.<br><br>This statement is required for submission and **will appear in the published article** if the submission is accepted. Please make sure it is accurate.	Financial support provided by Research and Innovation University Grant Scheme, Universiti Teknologi Malaysia, is gratefully acknowledged

Powered by Editorial Manager® and ProduXion Manager® from Aries Systems Corporation

**Unfunded studies**<br>Enter: *The author(s) received no specific funding for this work.*<br><br>**Funded studies**<br>Enter a statement with the following details:<br>• Initials of the authors who received each award<br>• Grant numbers awarded to each author<br>• The full name of each funder<br>• URL of each funder website<br>• Did the sponsors or funders play any role in the study design, data collection and analysis, decision to publish, or preparation of the manuscript?<br>• **NO** - Include this sentence at the end of your statement: *The funders had no role in study design, data collection and analysis, decision to publish, or preparation of the manuscript.*<br>• **YES** - Specify the role(s) played.<br><br>* typeset	
**Competing Interests**<br><br>Use the instructions below to enter a competing interest statement for this submission. On behalf of all authors, disclose any competing interests that could be perceived to bias this work—acknowledging all financial support and any other relevant financial or non-financial competing interests.<br><br>This statement **will appear in the published article** if the submission is accepted. Please make sure it is accurate. View published research articles from *PLOS ONE* for specific examples.	The authors declare that there is no conflict of interests regarding the publication of this paper

**NO authors have competing interests**<br><br>Enter: *The authors have declared that no competing interests exist.*<br><br>**Authors with competing interests**<br><br>Enter competing interest details beginning with this statement:<br><br>*I have read the journal's policy and the authors of this manuscript have the following competing interests: [insert competing interests here]*<br><br>* typeset	
**Ethics Statement**<br><br>Enter an ethics statement for this submission. This statement is required if the study involved:<br><br>• Human participants<br>• Human specimens or tissue<br>• Vertebrate animals or cephalopods<br>• Vertebrate embryos or tissues<br>• Field research<br><br>Write "N/A" if the submission does not require an ethics statement.<br><br>General guidance is provided below. Consult the submission guidelines for detailed instructions. **Make sure that all information entered here is included in the Methods section of the manuscript.**	The authors declare that there is no conflict of interests regarding the publication of the manuscript. It has not been published before nor submitted to any other journal for the consideration of publication.

**Format for specific study types**<br><br>**Human Subject Research (involving human participants and/or tissue)**<br>• Give the name of the institutional review board or ethics committee that approved the study<br>• Include the approval number and/or a statement indicating approval of this research<br>• Indicate the form of consent obtained (written/oral) or the reason that consent was not obtained (e.g. the data were analyzed anonymously)<br><br>**Animal Research (involving vertebrate animals, embryos or tissues)**<br>• Provide the name of the Institutional Animal Care and Use Committee (IACUC) or other relevant ethics board that reviewed the study protocol, and indicate whether they approved this research or granted a formal waiver of ethical approval<br>• Include an approval number if one was obtained<br>• If the study involved *non-human primates*, add *additional details* about animal welfare and steps taken to ameliorate suffering<br>• If anesthesia, euthanasia, or any kind of animal sacrifice is part of the study, include briefly which substances and/or methods were applied<br><br>**Field Research**<br><br>Include the following details if this study involves the collection of plant, animal, or other materials from a natural setting:<br>• Field permit number<br>• Name of the institution or relevant body that granted permission	
**Data Availability**<br><br>Authors are required to make all data underlying the findings described fully available, without restriction, and from the time of publication. PLOS allows rare exceptions to address legal and ethical concerns. See the PLOS Data Policy and FAQ for detailed information.	Yes - all data are fully available without restriction

A Data Availability Statement describing where the data can be found is required at submission. Your answers to this question constitute the Data Availability Statement and **will be published in the article**, if accepted.<br><br>**Important:** Stating 'data available on request from the author' is not sufficient. If your data are only available upon request, select 'No' for the first question and explain your exceptional situation in the text box.<br><br>Do the authors confirm that all data underlying the findings described in their manuscript are fully available without restriction?	
**Describe where the data may be found in full sentences. If you are copying our sample text, replace any instances of XXX with the appropriate details.**<br><br>• If the data are **held or will be held in a public repository**, include URLs, accession numbers or DOIs. If this information will only be available after acceptance, indicate this by ticking the box below. For example: *All XXX files are available from the XXX database (accession number(s) XXX, XXX.*).<br>• If the data are all contained **within the manuscript and/or Supporting Information files**, enter the following: *All relevant data are within the manuscript and its Supporting Information files.*<br>• If neither of these applies but you are able to provide **details of access elsewhere**, with or without limitations, please do so. For example:<br><br>*Data cannot be shared publicly because of [XXX]. Data are available from the XXX Institutional Data Access / Ethics Committee (contact via XXX) for researchers who meet the criteria for access to confidential data.*<br><br>*The data underlying the results presented in the study are available from (include the name of the third party*	After acceptance of paper we will send all programs.

and contact information or URL).<br>• This text is appropriate if the data are owned by a third party and authors do not have permission to share the data.<br><br>* typeset	
Additional data availability information:	Tick here if the URLs/accession numbers/DOIs will be available only after acceptance of the manuscript for publication so that we can ensure their inclusion before publication.; Tick here if your circumstances are not covered by the questions above and you need the journal's help to make your data available.

Powered by Editorial Manager® and ProduXion Manager® from Aries Systems Corporation

Click here to download Manuscript Paper.docx

Controlling optimal pressure of hydrogen-natural gas mixture in an inclined pipeline

Sarkhosh S. Chaharborj, Norsarahaida Amin*

Department of Mathematical Sciences, Faculty of Science, Universiti Teknologi Malaysia, 81310 UTM Johor Bahru, Malaysia

sseddighi2014@yahoo.com.my, norsarahaida@utm.my

Abstract

This paper discusses the control of optim[illegible]ressure using the zero-gradient control (ZGC) approach. It is applied for the first time in the study to control the optimal pressure of hydrogen natural gas mixture in an inclined pipeline. The solution to the flow problem is first validated with existing results using the Taylor series approximation, regression analysis and the Runge-Kutta method combined. The optimal pressure is then determined using ZGC where the optimal set points are calculated without having to solve the non-linear system of equations associated with the standard optimization problem. It is shown that the mass ratio is the more effective parameter compared to the initial pressure in controlling the maximum variation of pressure in a gas pipeline.

Keywords: hydrogen, natural gas, gas mixture, Taylor series, regression analysis, Zero Gradient Control

AMS classification: 35Qxx, 65Kxx, 76Nxx

1. Introduction

Many petroleum companies utilize the existing natural gas pipeline networks to transport hydrogen and natural gas in the same pipeline to reduce transportation cost [1]. Gas pipeline networks are systems with hundreds or thousands of kilometers of pipes which contain compression stations and other devices like valves. These types of systems work at high pressure and use compression stations to supply gas over long distances [2].

Pressure change in pipelines depends on gas velocity, valve closure time, and arrangement of the closing valve [3]. Maximum pressure can occur during valve closure or at the end of the closure operation. Short times during valve closure are important in reducing the maximum pressure, especially in emergency situations. However, this pressure transient is difficult to control because the damage is not always visible until long after the event. Therefore, controlling transient pressure in a pipeline with respect to certain efficient parameters such as initial pressure and mass ratio are very important [4, 5].

The transient flow in hydrogen-natural gas mixtures ~~have~~ been studied by [2, 6, 7, 8]. They assumed that the flow is isothermal and the pipeline is laid horizontally. However, in reality most gas pipelines are not horizontal [9] studied the gas flow of mixed hydrogen with natural gas and transport via high pressure in an inclined pipeline. By reason of the gas properties can be assumed to be varied or not ~~constant~~ over any cross section in a pipeline one-dimensional, non-isothermal gas flow model was solved to simulate the slow and fast fluid transients, such as those typically found in high-pressure gas transmission pipelines in [2].

Analysis on mass ratio and pressure variation by developing numerical models and computer algorithms in paper [10, 11]. Controlling of transient gas flow in complex pipeline intersection areas has been studied using a linear approximation of the equations describing the physics of gas flow in pipelines and formulated the optimization problem as a mixed-integer program in the paper [12]. A computer control algorithm has been used to study optimization of gas networks under transient conditions in the paper [13]. In the paper [14], a model is proposed to predict the decompression wave speed of high-pressure hydrogen-natural gas mixtures in pipelines. In the papers [10, 11, 12, 13, 14], controlling the high pressure and transient pressure have been studied using modified computer algorithms and numerical methods.

In paper [15] a method called Zero Gradient Control (ZGC) was proposed for controlling transient pressure, which is used to control the heat rejection pressure and the rotational speed of the fans of the gas cooler of R-744 refrigeration cycles nearly energy optimal. It combines ideas of Extremum Seeking Control (ESC) [16] and the online algorithms [17, 18, 19].

In the present paper, the Taylor series, regression analysis and zero gradient control have been applied to control the transient pressure. This is the first time, ZGC method is applied in hydrogen natural gas mixture in an inclined pipeline to control transient pressure. The optimal pressure is then determined using ZGC where the optimal set points are calculated without

having to solve the non-linear system of equations associated with the standard optimization problem.

Remind that, solving the equations of hydrogen-natural gas mixtures in pipelines without assuming the celerity pressure wave as constant is more complicated and cannot be solved in the any software's. To overcome these difficultie[illegible]n use the Taylor series of celerity pressure wave. Approximated series for density and celerity pressure wave will be proposed using the Taylor series. The zero gradient will have applied to approximated series to control the transient pressure. We will study the effect of initial pressure and mass ratio on the maximum values of transient pressure. Effect of initial pressure and transient pressure on the optimal values of mass ratio will study to control the transient pressure.

2. Mathematical Formulation

According references [2, 6], a single inclined pipeline with a compressor at the upstream end and a sudden closure valve at the downstream end is considered see Fig.(1). The governing equations consist of three coupled non-linear hyperbolic partial differential equations. The flow is one dimensional, non-isothermal, and compressible. The fluid is a homogeneous mixture of hydrogen and natural gas.

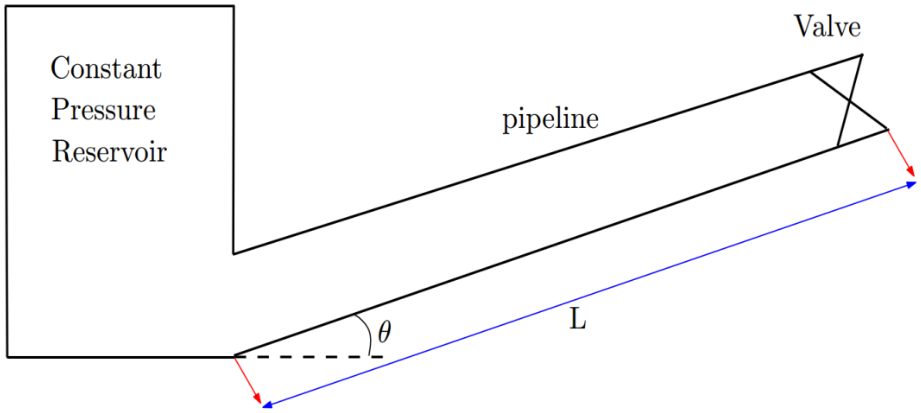

Fig. 1: The system consists of a simple inclined pipeline with a reservoir upstream and a valve downstream of the pipeline.

2.1. Governing equation

From references [6, 30] the principle of conservation of mass and momentum laws, the governing equations and the initial and boundary conditions for the transportation of hydrogen-natural gas mixture in an inclined pipeline and using the relation $P = c^2\rho$ can be written as,

$$\frac{\partial P}{\partial t} + \frac{\partial Pu}{\partial x} = 0, \tag{1}$$

$$\frac{\partial Pu}{\partial t} + \frac{\partial (Pu^2 + c^2)}{\partial x} + \frac{fPu|u|}{2D} + Pg\sin\theta = 0, \tag{2}$$

with the boundary conditions as follows,

$$P(0,t) = P_0(t), u(0,t) = u_0(t), \quad \text{(at the initial point, } x = 0\text{)}, \tag{3}$$

$$P(L,t) = P(t), u(L,t) = u_L(t), \quad \text{(at the initial point, } x = L\text{)}, \tag{4}$$

with $u_0 = Q_0/A$ and $A = \pi D^2/4$, where ρ is defined as density, u is the gas velocity where the modulus sign is to ensure that the frictional force shall always act opposite to the direction of motion, P is the pressure, e is the gas internal energy per unit mass, f is the coefficient of friction, D is the diameter of the pipeline, g is the gravitational force and θ is an angle between the friction force and the x direction.

The equation of state for perfect gas, which is commonly used in the gas industry, is given by, $P = \rho RT$, where R is the specific gas constant and T is the temperature. For compressible flow, the relation of equation of state with the celerity pressure wave c is denoted as, $P = c^2\rho$. From relation of perfect gas, the specific heat at constant volume C_v, the specific heat at constant pressure C_P, the specific gas constant R, pressure P and flow process index γ are defined as,

$$C_p - C_v = R, \quad \gamma = \frac{C_p}{C_v}, \; C_v = \frac{R}{\gamma - 1}.$$

2.2. Hydrogen-natural gas mixture equation

The mass ratio and the density of hydrogen-natural gas mixture are defined as,

$$\phi = \frac{m_h}{m_h + m_g}, \qquad \frac{1}{\rho} = \frac{v_h + v_g}{m_h + m_g},$$

with $v_h = \frac{m_h}{\rho_h}, v_g = \frac{m_g}{\rho_g}, \rho_h = \rho_{h_0}\left(\frac{P}{P_0}\right)^{\frac{1}{n_1}}, \rho_g = \rho_g\left(\frac{P}{P_0}\right)^{\frac{1}{n_2}}$.

therefore, the expression of the average density of the gas mixture is given by,

$$\rho = \left[\frac{\phi}{\rho_{h_0}}\left(\frac{P_0}{P}\right)^{\frac{1}{n_1}} + \frac{1-\phi}{\rho_{g_0}}\left(\frac{P_0}{P}\right)^{\frac{1}{n_2}}\right]^{-1}. \tag{5}$$

For compressible flow, the celerity pressure wave is defined as,

$$c = \left(\frac{\partial \rho}{\partial P}\right)_s^{-\frac{1}{2}}, \tag{6}$$

where the subscript s denotes the condition of constant entropy. By taking the derivative of equation (5) with respect to P, and substituting into equation (6), then the celerity pressure wave yields [6, 30],

$$c = \left[\frac{\phi}{\rho_{h_0}}\left(\frac{P_0}{P}\right)^{\frac{1}{n_1}} + \frac{1-\phi}{\rho_{g_0}}\left(\frac{P_0}{P}\right)^{\frac{1}{n_2}}\right] \times \left[\frac{1}{P}\left[\frac{\phi}{n_1\rho_{h_0}}\left(\frac{P_0}{P}\right)^{\frac{1}{n_1}} + \frac{1-\phi}{n_2\rho_{g_0}}\left(\frac{P_0}{P}\right)^{\frac{1}{n_2}}\right]\right]^{-\frac{1}{2}}. \tag{7}$$

The properties of hydrogen and natural gas used in the calculations are ~~showed~~ in the Tables (1). For simulation ~~calculation and obtaining the numerical results~~ the ~~known~~ parameters are assumed as Table (2).

Table 1: Hydrogen properties in working conditions, $P = 35$ bar and T = 15 $^{\circ}$C=288 K [6, 30].

Symbol	Fluid properties	Values (J/kgK)	
		Hydrogen	Natural gas
C_p	Specific heat at constant pressure	14600	1497.5
C_v	Specific heat at constant volume	10440	1056.8
R	Gas constant	4160	440.7

Table 2: ~~Known~~ parameters for simulation calculation and obtaining the numerical results [6, 30].

Symbols	Values	Symbols	Values
Pipe length	L=600 m	Mass ratio	$\phi = 0, 0.5, 1$
Time	t=20	Angle	$\theta = 0, \pi/6, \pi/4, \pi/3$
Pipe diameter	D=0.4 m	Initial mass flow	Q_0 =55 kg/s
Friction coefficient	$f = 0.03$	Initial absolute pressure	$P_0 = 35$ bar
Temperature	$T = 15$ °C=288 K		

3. Approximate Series for Density and Celerity

In this section, we use the Taylor series expansion as presented in the reference [31] for finding the approximate series of equations (5) and (7).

3.1.Approximate series for density

Because of the singularity point $P = 0$, to find the Taylor series of equation (5) is not possible, to overcome this problem we can use e^P instead of P,

$$\rho(e^P) = \left[\frac{\phi}{\rho_{h_0}}\left(\frac{P_0}{e^P}\right)^{\frac{1}{n_1}} + \frac{1-\phi}{\rho_{g_0}}\left(\frac{P_0}{e^P}\right)^{\frac{1}{n_2}}\right]^{-1}. \tag{8}$$

then the Taylor series of equation (8) around point $P = 0$ from order 2 is given as follows,

$$\rho(e^P) \simeq [\rho(e^P)]_{P=0} + \frac{P}{1!}[\rho(e^P)]'_{P=0} + \frac{P^2}{2!}[\rho(e^P)]''_{P=0},$$

after replacing the derivatives $[\rho(e^P)]'_{P=0}$ and $[\rho(e^P)]''_{P=0}$ we ~~will~~ have~~,~~

$$\rho(e^P) \simeq \frac{1}{A_3}\left[1 - \frac{A_4}{A_6}\rho_{h_0}\rho_{g_0}P - \frac{\rho_{h_0}\rho_{g_0}}{n_1 n_2 A_6^2}[n_1 n_2 A_5 A_6 + A_4 A_7]P^2\right],$$

with $A_1, A_2, \dots, A_7$ ~~are~~ defined as follows~~,~~

$$A_1 = e^{\frac{\ln(P_0)}{n_1}}, \quad A_2 = e^{\frac{\ln(P_0)}{n_2}},$$
$$A_3 = \frac{\phi}{\rho_{h_0}}A_1 + \frac{1-\phi}{\rho_{g_0}}A_2, \; A_4 = -\frac{\phi}{\rho_{h_0 n_1}}A_1 - \frac{1-\phi}{\rho_{g_0} n_2}A_2,$$
$$A_5 = \frac{\phi}{2\rho_{h_0 n_1^2}}A_1 + \frac{1-\phi}{2\rho_{g_0} n_2^2}A_2, \; A_6 = \phi\rho_{g_0}A_1 + (1-\phi)\rho_{h_0}A_2,$$
$$A_7 = n_2\phi\rho_{g_0}A_1 + n_1(1-\phi)\rho_{h_0}A_2,$$

now the function $\rho(e^P)$ is ~~converting~~ to function $\rho(P)$, in equation (9) by replacing P with $\ln(P)$,

$$\rho(e^P) \simeq \frac{1}{A_3}\left[1 - \frac{A_4}{A_6}\rho_{h_0}\rho_{g_0}[\ln P] - \frac{\rho_{h_0}\rho_{g_0}}{n_1 n_2 A_6^2}[n_1 n_2 A_5 A_6 + A_4 A_7][\ln P]^2\right]. \qquad (9)$$

Fig. (2) shows a comparison of the density evolution with pressure between exact solution (5) and approximation series (9), for different values of the hydrogen mass fraction ϕ, by assuming an initial pressure $P_0 = 35$ bar and $T_0 = 15\ ^{\circ}C = 288\ K$.

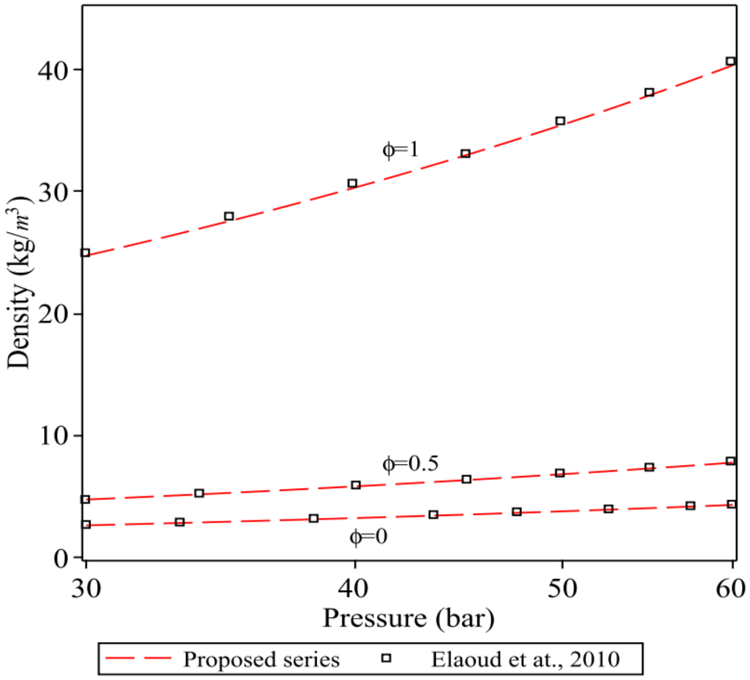

Figure 2: Density as a function of the pressure for different values of ϕ.

3.2. Approximate series for celerity pressure wave

To find the Taylor series of equation (7) we follow the presented method in Section (3.1),

$$c(e^P) \simeq \left[\frac{\phi}{\rho_{h_0}}\left(\frac{P_0}{e^P}\right)^{\frac{1}{n_1}} + \frac{1-\phi}{\rho_{g_0}}\left(\frac{P_0}{e^P}\right)^{\frac{1}{n_2}}\right] \times \left[\frac{1}{P}\left[\frac{\phi}{n_1\rho_{h_0}}\left(\frac{P_0}{e^P}\right)^{\frac{1}{n_1}} + \frac{1-\phi}{n_2\rho_{g_0}}\left(\frac{P_0}{e^P}\right)^{\frac{1}{n_2}}\right]\right]^{-\frac{1}{2}}. \qquad (10)$$

then the Taylor series of equation (10) around point $P = 0$ from order 2 is given as follows,

$$c(e^P) \simeq [c(e^P)]_{P=0} + \frac{P}{1!}[c(e^P)]'_{P=0} + \frac{P^2}{2!}[c(e^P)]''_{P=0},$$

after replacing the derivatives $[c(e^P)]'_{P=0}$ and $[c(e^P)]''_{P=0}$ we ~~will~~ have~~,~~

$$
\begin{aligned}
c\left(e^{P}\right) \simeq & \left[\frac{\rho_{h_0}\rho_{g_0}\left[\phi\rho_{g_0}n_2C_1-\rho_{h_0}n_1\phi C_2+\rho_{h_0}n_1C_2\right]}{n_1n_2\left[\phi\rho_{g_0}C_1-\rho_{h_0}\phi C_2+\rho_{h_0}C_2\right]^2}\right]^{-\frac{1}{2}} \\
& -\frac{C_8C_{12}P}{2C_6C_7n_1\sqrt{n_2}}+\frac{C_{13}P^2}{8C_6{C_7}^2\sqrt{C_8}{n_1}^2{n_2}^2},
\end{aligned}
\tag{11}
$$

with $C_1, C_2, \ldots, C_{13}$ are defined as follows,

$$
C_1 = {P_0}^{\frac{1}{n_1}},\ C_2 = {P_0}^{\frac{1}{n_2}},\ C_3 = {P_0}^{\frac{n_2+2\,n_1}{n_1n_2}},\ C_4 = {P_0}^{\frac{n_2+n_1}{n_1n_2}},\ C_5 = {P_0}^{\frac{2\,n_2+n_1}{n_1n_2}},
$$

$$
C_6 = \rho_{h_0}\left(1-\phi\right)C_2+\phi\,\rho_{g_0}C_1,\ C_7 = n_1\rho_{h_0}\left(\phi-1\right)C_2-\phi\rho_{g_0}n_2C_1,
$$

$$
C_8 = -\frac{\rho_{g_0}\rho_{h_0}C_7}{n_1n_2C_6^2},\ C_9 = \left[1+n_2\right]{n_1}^2+\left[{n_2}^2-4\,n_2\right]n_1+{n_2}^2,
$$

$$
\begin{aligned}
C_{10} = & \left[2\,{n_1}^2+2\,n_1-2\right]{n_2}^3+\left[{n_1}^3-10\,{n_1}^2+6\,n_1\right]{n_2}^2 \\
& +\left[2\,{n_1}^3-2\,{n_1}^2\right]n_2+{n_1}^3
\end{aligned}
$$

$$
C_{11} = \frac{1}{2}\left[n_1+1\right]^2{n_2}^3+\left[{n_1}^3-5\,{n_1}^2-n_1\right]{n_2}^2+{n_1}^2\left[n_1+3\right]n_2-{n_1}^3,
$$

$$
\begin{aligned}
C_{12} = & \rho_{g_0}\rho_{h_0}\phi\left(1-\phi\right)C_4C_9+{n_1}^2\left(n_2-1\right){\rho_{h_0}}^2\left(\phi-1\right)^2C_2^2 \\
& +\left(n_1-1\right){n_2}^2{\rho_{g_0}}^2\phi^2C_1^2,
\end{aligned}
$$

$$
\begin{aligned}
C_{13} = & n_1\rho_{g_0}{\rho_{h_0}}^2\phi\left(\phi-1\right)^2C_3C_{10}-2n_2{\rho_{g_0}}^2\rho_{h_0}\phi^2\left(\phi-1\right)C_5C_{11} \\
& +{n_2}^4\left(n_1-1\right)^2{\rho_{g_0}}^3\phi^3C_1^3-{n_1}^4\left(n_2-1\right)^2{\rho_{h_0}}^3\left(\phi-1\right)^3C_2^3.
\end{aligned}
$$

in equation (11) the function $\rho(e^P)$ can convert to function $\rho(P)$, by replacing P with $\ln(P)$,

$$
\begin{aligned}
c(P) \simeq & \left[\frac{\rho_{h_0}\rho_{g_0}\left[\phi\rho_{g_0}n_2C_1-\rho_{h_0}n_1\phi C_2+\rho_{h_0}n_1C_2\right]}{n_1n_2\left[\phi\rho_{g_0}C_1-\rho_{h_0}\phi C_2+\rho_{h_0}C_2\right]^2}\right]^{-\frac{1}{2}} \\
& -\frac{C_8C_{12}\left[\ln(P)\right]}{2C_6C_7n_1\sqrt{n_2}}+\frac{C_{13}\left[\ln(P)\right]^2}{8C_6{C_7}^2\sqrt{C_8}{n_1}^2{n_2}^2},
\end{aligned}
\tag{12}
$$

Fig. (3) shows a comparison of the density evolution with pressure between exact solution (7) and approximation series (12), for different values of the hydrogen mass fraction ϕ, by assuming an initial pressure P0 = 35 bar and T0 = 15 ◦C = 288 K.

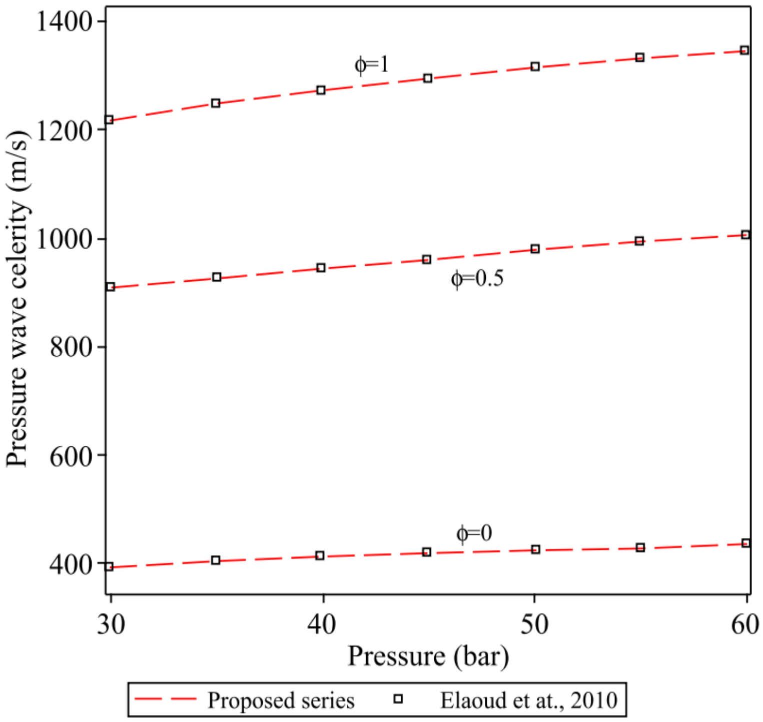

Figure 3: Celerity pressure wave as a function of the pressure for different values of ϕ.

4. Solution of Hydrogen-Natural Gas Mixture Equations

In this Section at first, we can use the Runge-Kutta method to solve the steady state equations. Regression analysis will apply to find the regression polynomials for pressure P and velocity u from the numerical results. Regression polynomials can use in solving hydrogen-natural gas mixture equations.

4.1. Solving steady state equations

Steady state equations can obtain from equations (1)-(2) with replacing t = 0 as follows,

$$\frac{d(Pu)}{dx} = 0, \tag{13}$$

$$\frac{d(Pu^2)}{dx} + c^2\frac{dP}{dx} + \frac{fPu|u|}{2D} + Pg\sin\theta = 0, \tag{14}$$

with the boundary conditions,

$$u(0,0) = u(0), \;\; p(0,0) = P(0) \tag{15}$$

Equations (13)-(14) can be solved using the Runge-Kutta method as numerically. Next use the regression analysis to find the regression polynomials for pressure P and velocity u as,

$$P(x) = a_0 + a_1 x + a_2 x^2, \quad (16)$$

$$u(x) = b_0 + b_1 x + b_2 x^2, \quad (17)$$

The polynomials $P(x)$ and $u(x)$ will be used as initial conditions for solving equations (1)-(2). The unknown parameters a_0, a_1, a_2 and b_0, b_1, b_2 are found using the error function defined as~~,~~

$$E = \sum_{i=0}^{m} [f(x_i) - y_i]^2, \quad (17)$$

where, $f(x_i) = P(x_i)$ or $f(x_i) = u(x_i)$ and m is the number of partitions for the pipeline L. Here the values x_i are the pipeline partition points and $y_i = y_{P,i}$ (pressure) or $y_i = y_{u,i}$ (celerity) are generated data from solving equations (13)-(14). With replacing the polynomials $P(x_i)$ and $u(x_i)$ in the error function (17) we have~~,~~

$$E_P = \sum_{i=0}^{m} \left[a_0 + a_1 x + a_2 x^2 - y_{P,i}\right]^2 \quad (18)$$

$$E_u = \sum_{i=0}^{m} \left[b_0 + b_1 x + b_2 x^2 - y_{u,i}\right]^2. \quad (19)$$

We ~~look for the zero[illegible]adient of~~ the error functions E_P and E_u to find the unknown parameters a_0, a_1, a_2 and b_0, b_1, b_2 for different values of mass ration $\phi = 0, 0.5, 1$ (see Table (3)).

Table 3: Values of coefficients a_0, a_1, a_2 and b_0, b_1, b_2 for various ϕ in Eq. (11).

parameters	$\phi = 0$	$\phi = 0.5$	$\phi = 1$
a_0	34.99999945	34.99992670	34.99947059
a_1	-0.00043023	-0.00215230	-0.004032469
a_2	0.0000000034598	0.0000000908154	0.0000003404764
b_0	143.23945768	143.24069650	143.24866196
b_1	0.00176059	0.00878971	0.01636548
b_2	0.0000000363624	0.000000989597	0.0000038470726

We have used Maple code to find the regression polynomials as follows,

```
sol:=dsolve({eq[1], eq[2], eq[3], ini}, numeric);
dX:=[seq(x, x=0..L, h)]:
P1:=[seq(abs(rhs(sol(x)[2])), x=0..L, h)]:
u1:=[seq(abs(rhs(sol(x)[3])), x=0..L, h)]:
P:=Fit(a1+a2*x+a3*x^2,TT2,P1,x);
u:=Fit(a1+a2*x+a3*x^2,TT2,u1,x);
```

with $eq[1] = Eq.(13)$, $eq[2] = Eq.(14)$, $ini = Eq.(15)$, L is pipe length and h is pipe length partition size.

Fig. (4) shows a validation between the regression polynomials of pressure $P(x)$ (equations (16)) with the numerical results of Elaoud et al. and Subani et al. papers as presented in the references [30, 3] for different values of mass ratio $\phi = 0, 0.5, 1$.

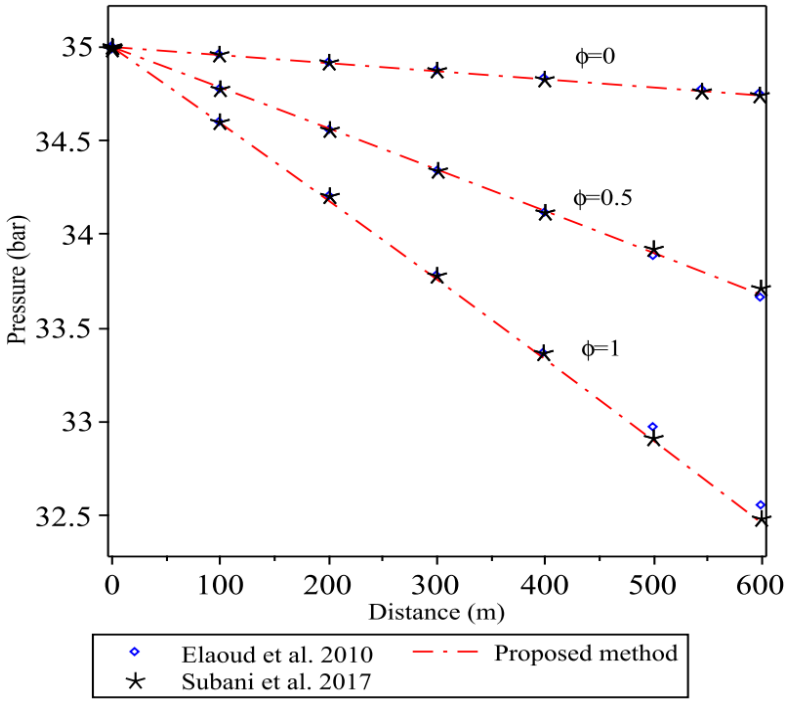

Figure 4: Permanent regime pressure distribution along the pipe (equations (13) and (14)) for different mass ratio $\phi = 0, 0.5, 1$.

4.2. Solving equations (1)-(2)

Now, by using the approximation series (12) and regression polynomials (16)-(17l) with coefficients as Table (3), we can solve the equations (1)-(2) by the following Maple package,

$$pdsolve(\{eqs\}, \{ini\}, numeric, time = t, range = 0..L, timestep = dt);$$

where, $eqs = \{Eq.(1),\ Eq.(2)\}$, $ini = \{Eq.(3),\ Eq.(4)\}$ and L is pipeline length.

Fig.(5)(a), (b) and (c) shows the numerical results for the transient pressure distribution after rapid closure of the downstream valve as a function of time and for different values of the hydrogen mass ratio ϕ. The results show that the obtained results using proposed method are in

good agreement with those of reduced order modelling (ROM) presented by Subani et al. in the paper [8] and the method of characteristics (MOC) presented by Elaoud at al. in the paper [30]. Then, our method is working as well as other methods and give the smoother results.

The transient pressure distribution for the rapid closure valve at the downstream end of the pipe for different values of $\theta = 0, \pi/6, \pi/4, \pi/3$ and mass ratio $\phi = 0.5$ are presented in Fig.(6)(a), where in the gas mixture ($\phi = 0.5$), increasing parameter angle θ from 0 to $\pi/3$, will increase the transient pressure. The minimum and maximum transient pressures will happen in the values of $\theta = 0$ and $\theta = \pi/3$, respectively. With increasing the mass ratio from 0 to 1, transient pressure is increasing.

Fig.(6)(b) indicates the transient pressure distribution for the rapid closure valve at the downstream end of the pipe for different values of mass ratio $\phi = 0, 0.5, 1$ and $\theta = 0$. As can be seen from Fig.(6)(b), transient pressure peaks for $\phi = 0, \phi = 0.5$ and $\phi = 1$ are 6, 4 and 2 respectively. With increasing the mass ratio from 0 to 1, transient pressure is increasing.

Fig.(6)(c) shows the transient pressure distribution for the rapid closure valve at the downstream end of the pipe for different values of permanent pressure $P_0 = 30, 60$ and $\theta = 0$, $\phi = 0.5$. Presented results in this figure are showing that the variation of permanent pressure P_0 don't have much effective in changing of transient pressure.

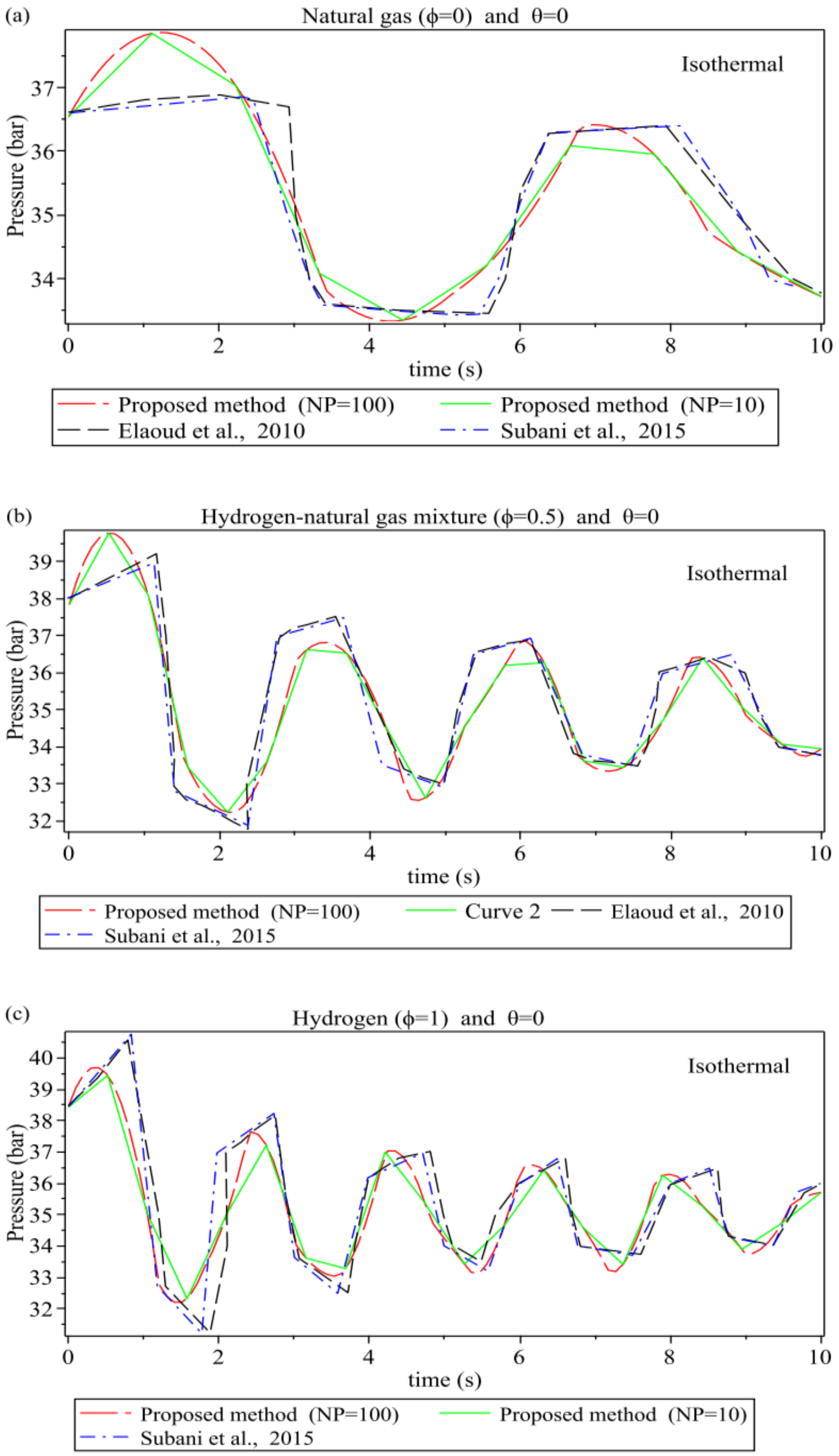

Figure 5: Transient pressure distribution for the rapid closure valve at the downstream end of the pipe for different values of mass ratio ϕ and $\theta = 0$ [8, 30].

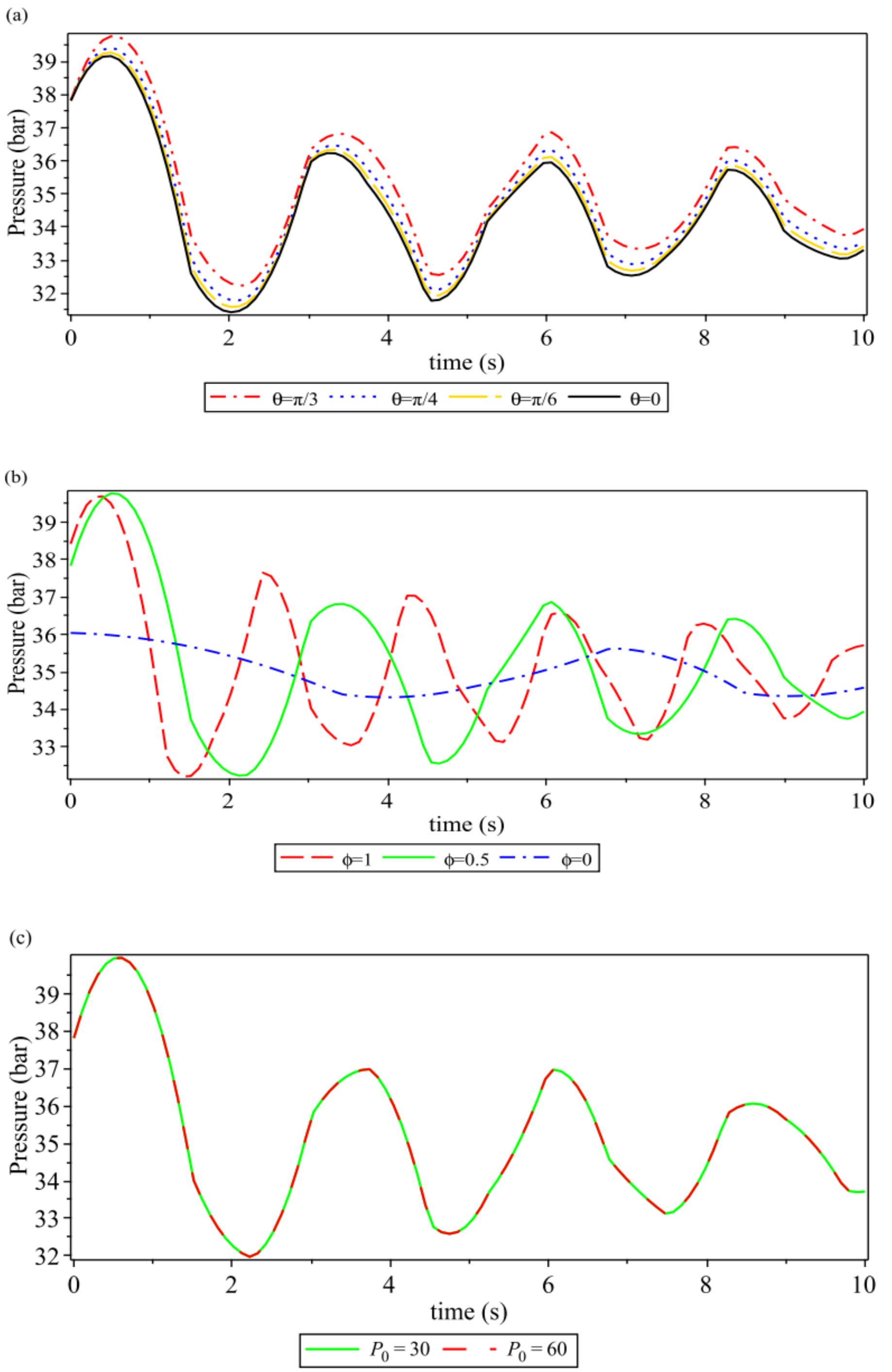

Figure 6: Transient pressure distribution for the rapid closure valve at the downstream end of the pipe. (a): for different values of $\theta = 0, \pi/6, \pi/4, \pi/3$ and mass ratio $\phi = 0.5$; (b): for different values of mass ratio $\phi = 0, 0.5, 1$ and $\theta = 0$; (c): for different values of permanent pressure $P_0 = 30, 60$ and $\theta = 0$, $\phi = 0.5$.

5. Zero Gradient Control for Controlling the Pressure

To find the optimum values of pressure P, we need to ~~use the zeros gradient~~ of the equations (5) with respect to the pressure P. But using the basic equation of ρ as proposed in the equation (5) cannot guarantee the proper optimization for the transient pressure (see reference [33]). To overcome this problem, we will use the Taylor series as shown in the equations (9). To find the optimal values of the function $\rho(P)$ with respect to the transient pressure P (optimal values of pressure), we look for the zeros gradient of the series (9) as follows,

$$\frac{\partial \rho}{\partial P} = \frac{\rho_{g_0}\rho_{h_0}\left[\left[A_2{}^2 n_1{}^2 (\phi-1)^2 \rho_{h_0}{}^2 + B_3 + A_1{}^2\phi^2 n_2{}^2 \rho_{g_0}{}^2\right] \ln(P) - n_1 n_2 B_1 B_2\right]}{n_1{}^2 n_2{}^2 P B_1{}^3}, \tag{20}$$

where B_1, B_2, B_3 are,

$B_1 = \phi A_1 \rho_{g_0} - A_2(\phi-1)\rho_{h_0}$,

$B_2 = A_2 n_1(\phi-1)\rho_{h_0} - \phi A_1 \rho_{g_0} n_2$,

$B_3 = A_1 A_2 \phi(\phi-1)\rho_{h_0}\rho_{g_0}[n_1^2 - 4n_1 n_2 + n_2^2]$,

solving equation $\frac{\partial\rho}{\partial P} = 0$ we can obtain the explicit formula for the optimal values of transient pressure P,

$$P_{\text{optimal},\rho} \simeq e^{\frac{n_1 n_2 B_1 B_2}{\phi^2 A_1{}^2 n_2{}^2 \rho_{g_0}{}^2 + \phi^2 A_2{}^2 n_1{}^2 \rho_{h_0}{}^2 - 2\phi A_2{}^2 n_1{}^2 \rho_{h_0}{}^2 + A_2{}^2 n_1{}^2 \rho_{h_0}{}^2 + B_3}}. \tag{21}$$

Now, to find the optimum values of $c(P)$ with respect to pressure P we look for the zeros gradient of the series (11), and by solving equation $\frac{\partial c}{\partial P} = 0$ we obtain the explicit formula for the optimal values of transient pressure as follows,

$$P_{\text{optimal,c}} \simeq e^{\frac{2n_1 n_2 C_7 C_{12}}{C_{13}}} \tag{22}$$

with C_7, C_{12} and C_{13} are defined as follows,

$$C_7 = n_1 \rho_{h_0}(\phi-1) C_2 - \phi\rho_{g_0} n_2 C_1,$$

$$C_{12} = \rho_{g_0}\rho_{h_0}\phi(1-\phi) C_4 C_9 + n_1{}^2 (n_2-1)\rho_{h_0}{}^2 (\phi-1)^2 C_2^2 + (n_1-1) n_2{}^2 \rho_{g_0}{}^2 \phi^2 C_1^2,$$

$$C_{13} = n_1 \rho_{g_0}\rho_{h_0}{}^2 \phi(\phi-1)^2 C_3 C_{10} - 2n_2 \rho_{g_0}{}^2 \rho_{h_0}\phi^2 (\phi-1) C_5 C_{11} + n_2{}^4 (n_1-1)^2 \rho_{g_0}{}^3 \phi^3 C_1^3 - n_1{}^4 (n_2-1)^2 \rho_{h_0}{}^3 (\phi-1)^3 C_2^3.$$

Fig.(7)(a) and (b) show the optimum values of the pressure P as a function of the initial pressure P_0 and as a function of the mass ratio ϕ, respectively; by using the equation (21) for $= 0.5$ and $P_0 = 35$. As seen in Fig.(7)(a), the optimum values of the pressure P decrease ϕ from 0.00091710 to 0.00091700 with increasing the initial pressure P_0 from 30 to 60.

The results of Fig.(7)(b) show that with increasing mass ratio ϕ from 0 to 1, the optimal values of the transient pressure P decrease from 0.00110 to 0.00090. Therefore, the variation of optimum pressure $\left(\nabla P_{\text{optimal}}\right)$ with respect to the parameters initial pressure P_0 and mass ratio ϕ respectively are, $\nabla P_{\text{optimal}}(P_0) = 1 \times 10^{-7}$ and $\nabla P_{\text{optimal}}(\phi) = 2 \times 10^{-4}$. The values of the optimal pressure variations show that sensitivity of the optimal values [27, 28, 29] of the transient pressure P with respect to the mass ratio ϕ is $\frac{\nabla P_{\text{optimal}}(\phi)}{\nabla P_{\text{optimal}}(P_0)} = \frac{2\times 10^{-4}}{1\times 10^{-7}} = 2000$ times more than the initial pressure P_0 (see Fig.(6)(b) and Fig.(6)(c)).

Fig.(8)(a) and (b) shows the optimal values of the pressure P as a function of the initial pressure P_0 and as a function of the mass ratio ϕ, respectively; by using the equation (21) for $\phi = 0.5$ and $P_0 = 35$. As seen in Fig.(8)(a), the optimal values of the pressure P increases from 0.24654[illegible] to 0.2465450 with increasing initial pressure P_0 from 30 to 60. The results from Fig.(8)(b) shows that with increasing the mass ratio ϕ from 0 to 1, the optimum values of the pressure P increases from 0.2425 to 0.2470. For Fig.(8)(a) and (b) we have, $\nabla P_{\text{optimal}}(P_0) = 2.5 \times 10^{-6}$, $\nabla P_{\text{optimal}}(\phi) = 4.5 \times 10^{-3}$ and $\frac{\nabla P_{\text{optimal}}(\phi)}{\nabla P_{\text{optimal}}(P_0)} = \frac{2\times 10^{-3}}{1\times 10^{-6}} = 1800$.

According to Figs.(7)(a), (8)(a), ratio of the minimum and maximum optimal pressures are ~~as,~~ $\frac{\text{optimal}\{P\}_{P_0=30}}{\text{optimal}\{P\}_{P_0=60}} \simeq 1.011$ and $\frac{\text{optimal}\{P\}_{P_0=30}}{\text{optimal}\{P\}_{P_0=60}} \simeq 0.99$, respectively. This means that the initial pressure ~~is a less effective in varying~~ the optimum pressure in the pipeline. Figs.(7)(b) and (8)(b) show that $\phi = 0.4$ is a critical point for the variation of optimum pressure and for $\phi \in [0, 0.4]$ the variation of optimal pressure is faster than $\phi \in [\,0.4, 1]$. We have $\frac{\text{optimal}\{P\}_{\phi=0}}{\text{optimal}\{P\}_{\phi=0.4}} \simeq 1.19$, $\frac{\text{optimal}\{P\}_{\phi=0.4}}{\text{optimal}\{P\}_{\phi=1}} \simeq 1.02$, $\frac{\text{optimal}\{P\}_{\phi=0}}{\text{optimal}\{P\}_{\phi=1}} \simeq 1.22$ and $\frac{\text{optimal}\{P\}_{\phi=0}}{\text{optimal}\{P\}_{\phi=0.4}} \simeq 0.9949$, $\frac{\text{optimal}\{P\}_{\phi=0.4}}{\text{optimal}\{P\}_{\phi=1}} \simeq 0.9943$. This means that~~,~~ the mass ratio is very important parameter for controlling the optimum pressure in the pipeline. The Fig.(7)(a), (b) and (8)(a), (b) show that the mass ratio is important

case for controlling the optimum pressure in the pipeline. Fig.(6)(b) and Fig.(6)(c) show the validation of the results showed in the Fig.(7) and (8).

In statistics, a confidence interval (CI) is a type of interval estimate, computed from the statistics of the observed data, that might contain the true value of an unknown parameter [34, 35, 36]. Therefore, the confidence interval for the optimum values of the pressure P is defined as $\left[\min_{P_0,\phi} P_{\text{optimal}}, \max_{P_0,\phi} P_{\text{optimal}}\right]$, where $\min_{P_0,\phi} P_{\text{optimal}}$ and $\max_{P_0,\phi} P_{\text{optimal}}$ are given by,

$$\min_{P_0,\phi} P_{\text{optimal}} = \min_{P_0,\phi}\left\{\min_{P_0,\phi}\{\text{optimal } P_\rho\}, \min_{P_0,\phi}\{\text{optimal } P_c\}\right\} \tag{23}$$

$$\max_{P_0,\phi} P_{\text{optimal}} = \max_{P_0,\phi}\left\{\max_{P_0,\phi}\{\text{optimal } P_\rho\}, \max_{P_0,\phi}\{\text{optimal } P_c\}\right\} \tag{24}$$

with $\max_{P_0}\left[\left(P_{\text{optimal}}\right)_c\right] = 0.246545$, $\min_{P_0}\left[\left(P_{\text{optimal}}\right)_\rho\right] = 0.0009170$ and $\max_{\phi}\left[\left(P_{\text{optimal}}\right)_c\right] = 0.2470$, $\min_{\phi}\left[\left(P_{\text{optimal}}\right)_\rho\right] = 0.00090$.

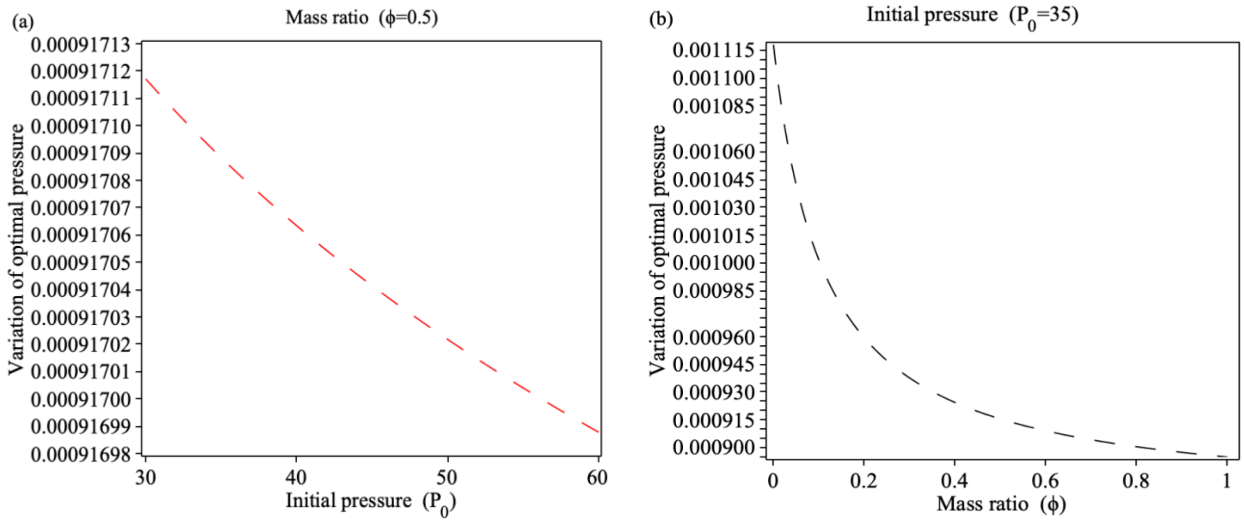

Figure 7: Variation of optimal pressure using equation (21); (a): as a function of the initial pressure P_0, (b): as a function of the mass ratio ϕ.

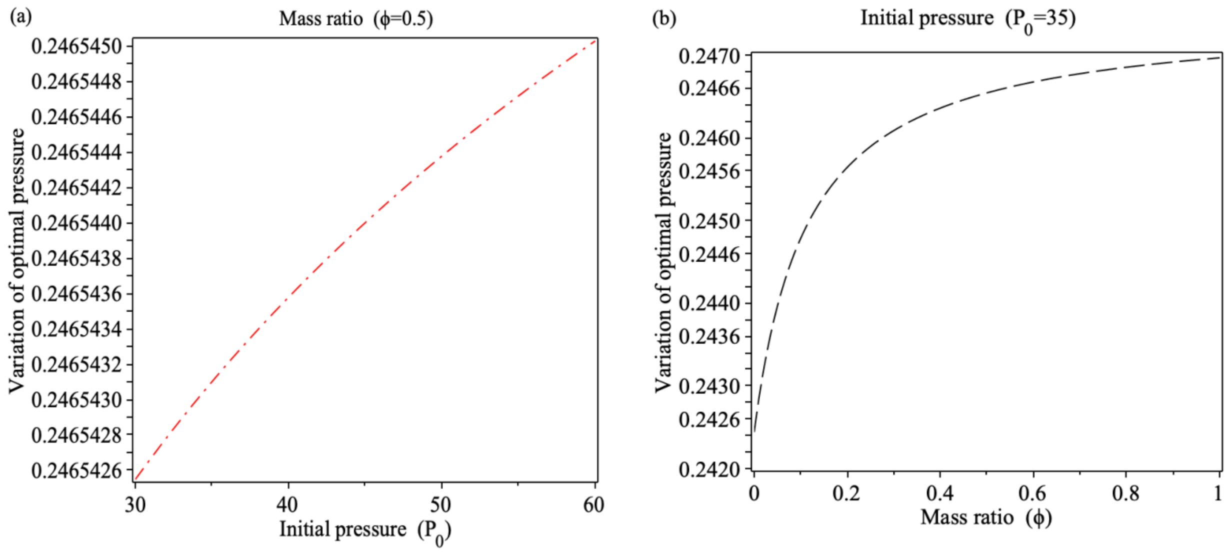

Figure 8: Variation of optimal pressure using equation (22); (a): as a function of the initial pressure P_0, (b): as a function of the mass ratio ϕ.

6. Pressure Control by Reining Mass Ratio

Taylor series and Zero Gradient Control are used here to control the mass ratio for obtaining efficient transient pressure. The optimum values for the mass ratio ϕ cannot be obtained using equations (5) and (7) to overcome this problem we use the Taylor series of those equations with respect to the parameter ϕ of order n as follows (see references [29, 31]),

$$\rho(\phi) = \rho(0) + \frac{\phi}{1!}\Big[\rho(\phi)\Big]'_{\phi=0} + \frac{\phi^2}{2!}\Big[\rho(\phi)\Big]''_{\phi=0} + \ldots + \frac{\phi^n}{n!}\Big[\rho(\phi)\Big]^{(n)}_{\phi=0} + ..,$$

$$c(\phi) = c(0) + \frac{\phi}{1!}\Big[c(\phi)\Big]'_{\phi=0} + \frac{\phi^2}{2!}\Big[c(\phi)\Big]''_{\phi=0} + \ldots + \frac{\phi^n}{n!}\Big[c(\phi)\Big]^{(n)}_{\phi=0} + \ldots,$$

for $n = 2$,

$$\rho(\phi) = \frac{\rho_{g0}}{L_2} - \frac{{\rho_{g0}}^2}{{L_2}^2}L_3\phi - \frac{{\rho_{g0}}^2\left(-L_1\rho_{g0} + L_2\rho_{h0}\right)}{{L_2}^3\rho_{h0}}L_3\phi^2, \qquad (25)$$

$$c(\phi) = \frac{1}{\sqrt{\frac{H_1\rho_{g0}}{n_2 P}}} - \frac{1}{2}\frac{(H_1 n_1 \rho_{h0} - 2\,H_2 n_1 \rho_{g0} + H_2 n_2 \rho_{g0})}{H_1 n_1 \rho_{h0}}.\frac{1}{\sqrt{\frac{H_1\rho_{g0}}{n_2 P}}}\phi + \frac{H_4}{\sqrt{\frac{H_1\rho_{g0}}{n_2 P}}}\phi^2, \tag{26}$$

with H_4,

$$H_4 = -\frac{1}{2}\frac{H_1{\rho_{h0}}^2 n_1 - 4\,H_2 n_1 \rho_{h0}\rho_{g0} + 2\,H_2\rho_{h0} n_2 \rho_{g0} + 3\,H_3 n_1 {\rho_{g0}}^2 - 2\,H_3 {\rho_{g0}}^2 n_2}{H_1{\rho_{h0}}^2 n_1} + \frac{3}{8}\frac{(H_1 n_1\rho_{h0} - 2\,H_2 n_1\rho_{g0} + H_2 n_2 \rho_{g0})^2}{{H_1}^2{n_1}^2{\rho_{h0}}^2}$$

and L_1, L_2, L_3 and H_1, H_2, H_3 are defined as,

$$L_1 = \left(\frac{P_0}{P}\right)^{\frac{1}{n_1}},\quad L_2 = \left(\frac{P_0}{P}\right)^{\frac{1}{n_2}},\quad L_3 = \frac{L_1}{\rho_{h0}} - \frac{L_2}{\rho_{g0}},$$

$$H_1 = \left(\frac{P_0}{P}\right)^{-\frac{1}{n_2}},\quad H_2 = \left(\frac{P_0}{P}\right)^{-\frac{2n_1-n_2}{n_1 n_2}},\quad H_3 = \left(\frac{P_0}{P}\right)^{-\frac{3n_1-2n_2}{n_1 n_2}}$$

To find the optimal values of $\rho(\phi)$ and $c(\phi)$ with respect to the parameter mass ratio ϕ, we look for the zeros of the gradient of the functions $\rho(\phi)$ and $c(\phi)$ as follows (see reference [33]),

$$\frac{\partial\rho}{\partial\phi} = -\frac{{\rho_{g0}}^2}{{L_2}^2}\left(\frac{L_1}{\rho_{h0}} - \frac{L_2}{\rho_{g0}}\right) - 2\,\frac{{\rho_{g0}}^2\,(-L_1\rho_{g0} + L_2\rho_{h0})\,\phi}{{L_2}^3\rho_{h0}}\left(\frac{L_1}{\rho_{h0}} - \frac{L_2}{\rho_{g0}}\right)$$

$$\frac{\partial c}{\partial\phi} = -\frac{1}{2}\frac{H_1 n_1\rho_{h0} - 2\,H_2 n_1\rho_{g0} + H_2 n_2\rho_{g0}}{H_1 n_1 \rho_{h0}}\frac{1}{\sqrt{\frac{H_1\rho_{g0}}{n_2 P}}} + 2\phi\frac{H_5}{\sqrt{\frac{H_1\rho_{g0}}{n_2 P}}}$$

with H_5 defined as follows,

$$H_5 = -\frac{1}{2}\frac{H_1{\rho_{h0}}^2 n_1 - 4\,H_2 n_1 \rho_{h0}\rho_{g0} + 2\,H_2\rho_{h0} n_2 \rho_{g0} + 3\,H_3 n_1 {\rho_{g0}}^2 - 2\,H_3 {\rho_{g0}}^2 n_2}{H_1{\rho_{h0}}^2 n_1} + \frac{3}{8}\frac{(H_1 n_1\rho_{h0} - 2\,H_2 n_1\rho_{g0} + H_2 n_2 \rho_{g0})^2}{{H_1}^2{n_1}^2{\rho_{h0}}^2},$$

and by solving the equations $\frac{\partial\rho}{\partial\phi} = 0$ and $\frac{\partial c}{\partial\phi} = 0$ we can obtain the optimal values of the mass ratio ϕ explicitly as follows,

$$\phi_{\mathrm{optimal},\rho} = \frac{1}{2}.\frac{L_2\rho_{h0}}{L_1\rho_{g0} - L_2\rho_{h0}}, \tag{27}$$

$$\phi_{\mathrm{optimal},c} = \frac{-2\Big[H_1 n_1\rho_{h0} - 2\,H_2 n_1 \rho_{g0} + H_2 n_2 \rho_{g0}\Big] n_1 \rho_{h0} H_1}{H_6}, \tag{28}$$

with H_6 defined as follows,

$$H_6 = {H_1}^2{n_1}^2{\rho_{h0}}^2 - 4\,H_1H_2{n_1}^2\rho_{g0}\rho_{h0} + 2\,H_1H_2n_1n_2\rho_{g0}\rho_{h0} + 12\,H_1H_3{n_1}^2{\rho_{g0}}^2 - 8\,H_1H_3n_1n_2{\rho_{g0}}^2 - 12\,{H_2}^2{n_1}^2{\rho_{g0}}^2 + 12\,{H_2}^2n_1n_2{\rho_{g0}}^2 - 3\,{H_2}^2{n_2}^2{\rho_{g0}}^2.$$

Fig.(9) presents the variation of optimal mass ratio ϕ as a function of the pressures P and P_0 by using the equations (25) and (26). Fig.(9)(a) and (b) show that the maximum and minimum values for the optimum mass ratio variations respectively are as, $\min_{\rho}\phi_{\text{optimal}} = 0.0708$, $\max_{\rho}\phi_{\text{optimal}} = 0.0725$ and $\min_{c}\phi_{\text{optimal}} = 0.288$, $\max_{c}\phi_{\text{optimal}} = 0.295$. As seen from the results with increasing the initial pressure P_0 from 30 up to 90, the variation optimum mass ratio increases. For $P = 60$, and different values of initial pressure P_0=30, 60, 90 we have the max mass ratio variations as $\Delta\phi_{\text{optimal,ρ}} = 0.07222, 0.07113, 0.07132$ $\Delta\phi_{\text{optimal,c}} = 0.2915, 0.2892, 0.2882$. This means that for controlling the optimal pressure we must increase ϕ or decrease initial pressure P_0.

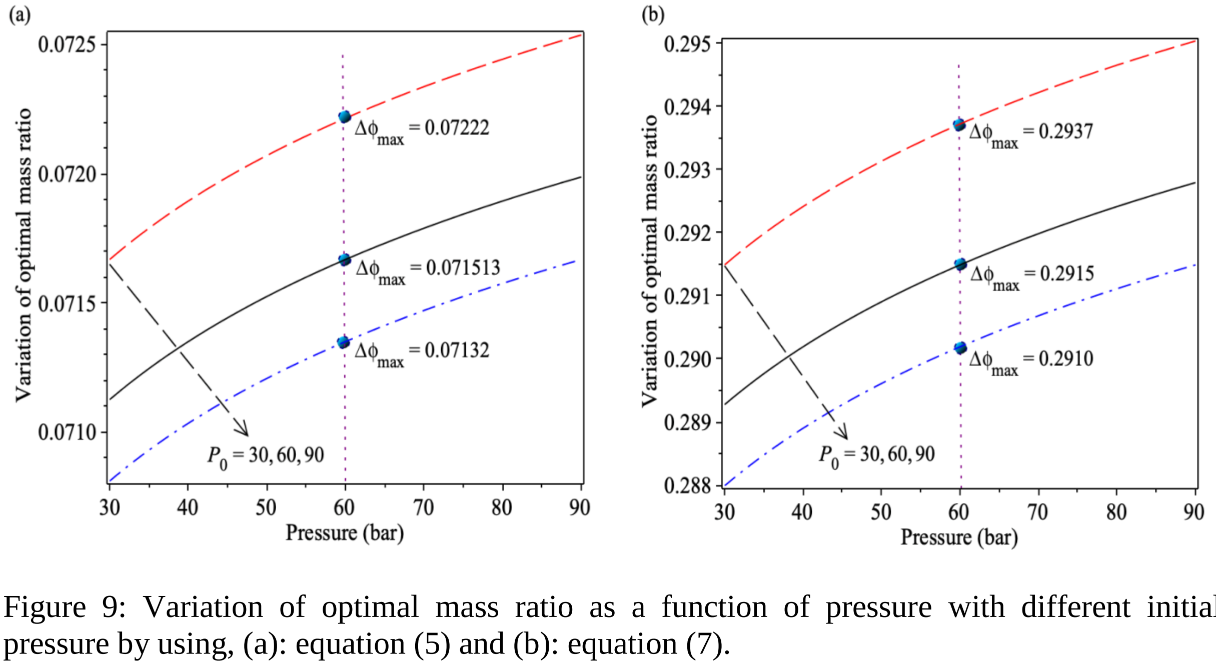

Figure 9: Variation of optimal mass ratio as a function of pressure with different initial pressure by using, (a): equation (5) and (b): equation (7).

7. Conclusion

The Taylor series approximation coupled with regression analysis used to solve the flow equations of hydrogen natural gas mixture in an inclined pipeline. To validate the approximation series, Fig. (3) shows a comparison of the density evolution with pressure between exact solution and approximation series, for different values of the hydrogen mass fraction ϕ, by assuming an initial pressure P0 = 35 bar and T0 = 15 ◦C = 288 K. Fig. (4) shows a validation between the regression polynomials of pressure $P(x)$ (equations (16)) with the numerical results of Elaoud et al. and Subani et al. papers for different values of mass ratio $\phi = 0, 0.5, 1$. The results in Fig.(5), (6) show that the obtained results using proposed method are in good agreement with those of reduced order modelling (ROM) and the method of characteristics (MOC). Then, our method is working as well as other methods and give the smoother results.

A method called Zero Gradient Control (ZGC) was applied to control the optimal transient pressure. The basic concept ZGC is to develop algebraic terms of density and celerity pressure wave equations for the partial derivatives with respect to initial pressure, mass ratio and transient pressure. A great advantage of the Zero Gradient Control is, that there is no need to calculate optimal set points, which would be required for solving a nonlinear system of equations. The equations derived for density and celerity pressure wave can be applied to many systems in different fields. According to the results of the proposed method the ratio of pressure with respect to the mass ratio ϕ and initial pressure P_0 are 1.22 and 1.011, respectively (see Figs.(7),(8)). The results show that the mass ratio is important for controlling the optimal pressure in an inclined pipeline. Fig.(9) shows that for $P = 60$, and different values of initial pressure P_0=30, 60, 90 we have the optimal mass ratio variations as $\Delta\phi_{\mathrm{optimal},\rho} = 0.07222, 0.07113, 0.07132$ and $\Delta\phi_{\mathrm{optimal,c}} = 0.2915, 0.2892, 0.2882$. This means that for controlling the optimal pressure we must increase ϕ or decrease initial pressure P_0.

Conflict of interests

The authors declare that there is no conflict of interests regarding the publication of this paper.

Acknowledgments

Financial support provided by Research and Innovation University Grant Scheme, Universiti Teknologi Malaysia, is gratefully acknowledged.

References

[1] Elaoud, S., Hadj-Taieb, E., Transient flow in pipelines of high-pressure hydrogen-natural gas mixtures. Int. J. Hydrogen Energy 33 (18) (2008), 4824-4832.

[2] Chaczykowski, M., Transient flow in natural gas pipeline the effect of pipeline thermal model. Appl. Math. Model. 34 (2010), 1051-1067.

[3] Karney, B.W., Ruus, E., Charts for water hammer in pipelines resulting from valve closure from full opening only. Canadian Journal of Civil Engineering, 12(2) (1985), 241-264.

[4] Kim, H., Kim, S., Kim, Y., Kim, J., Optimization of Operation Parameters for Direct Spring Loaded Pressure Relief Valve in a Pipeline System. Journal of Pressure Vessel Technology, 140(5) (2018), 051603.

[5] Jalving, J., Zavala, V. M., An Optimization-Based State Estimation Framework for Large-Scale Natural Gas Networks. Industrial & Engineering Chemistry Research, 57(17) (2018), 5966-5979.

[6] Subani, N., Amin, N., Agaie, B.G., Leak detection of non-isothermal transient flow of hydrogennatural gas mixture. Journal of Loss Prevention in the Process Industries 48 (2017), 244-253.

[7] Subani, N., Amin, N., Analysis of water hammer with different closing valve laws on transient flow of hydrogen-natural gas mixture. Abstr. Appl. Analysis 2015 (2015), 1-12.

[8] Subani, N., Amin, N., Agaie, B.G., Hydrogen-natural gas mixture leak detection using reduced order modelling. Appl. Comput. Math. 4 (3) (2015), 135-144.

[9] Uilhoorn, F.E., Dynamic behaviour of non-isothermal compressible natural gases mixed with hydrogen in pipelines. Int. J. Hydrogen Energy 34 (16) (2009), 6722-6729.

[10] Yu, T., Ding, Q., Wang, L., Zhu, D. Z., Shao, Y., Experimental study on air pressure variation in a horizontal pipe of single-stack drainage system. Water Science and Technology, 79(1) (2019), 114-125.

[11]Zaki, K., Imam, Y., El-Ansary, A., Optimizing the dynamic response of pressure reducing valves to transients in water networks. Journal of Water Supply: Research and Technology-Aqua (2019).

[12]Hennings, F., Anderson, L., Hoppmann, K., Turner, M., Koch, T., Controlling transient gas flow in complex pipeline intersection areas (2019).

[13]Osiadacz, A. J., Chaczykowski, M., Optimization of Gas Networks Under Transient Conditions. In PSIG Annual Meeting. Pipeline Simulation Interest Group, (2018).

[14]Liu, B., Liu, X., Lu, C., Godbole, A., Michal, G., Teng, L., Decompression of hydrogennatural gas mixtures in high-pressure pipelines: CFD modelling using different equations of state. International Journal of Hydrogen Energy, 44(14) (2019), 7428-7437.

[15]Noeding, M., Tegethoff, W., Koehler, J., Zero Gradient Control for R-744 Refrigeration Cycles. International Journal of Refrigeration, (2019).

[16]Hu, L., Xue, F., Qin, Z., Shi, J., Qiao, W., Yang, W., Yang, T., Sliding mode extremum seeking control based on improved invasive weed optimization for MPPT in wind energy conversion system. Applied Energy, 248 (2019), 567-575.

[17]Larsen, L. F. S., Thybo, C., Stoustrup, J., Rasmussen, H., A method for online steady state energy minimization, with application to refrigeration systems. In 2004 43rd IEEE Conference on Decision and Control (CDC)(IEEE Cat. No. 04CH37601) (Vol. 5, pp. 4708-4713) (2004, December). IEEE.

[18]Zhang, W. J., Zhang, C. L., A correlation-free on-line optimal control method of heat rejection pressures in CO2 transcritical systems. International Journal of Refrigeration, 34(4) (2011), 844-850.

[19]Kim, M. S., Kim, M. S., An on-line control method of optimal heat rejection pressure for CO2 refrigeration cycle. In Proceedings of the 10th IIR Gustav Lorentzen Conference on Natural Refrigerants, Delft, The Netherlands, Paper No. GL-303, (2012).

[20]Maxey, W. A., Fracture initiation, propagation, and arrest. In Proc. of Symposium on Line Pipe Research, (1974).

[21]Jensen, J. B., Skogestad, S., Optimal operation of simple refrigeration cycles: Part I: Degrees of freedom and optimality of sub-cooling. Computers & chemical engineering, 31(5-6) (2012), 712-721.

[22]Koeln, J. P., Alleyne, A. G., Optimal subcooling in vapor compression systems via extremum seeking control: Theory and experiments. International Journal of Refrigeration, 43 (2014), 14-25.

[23] Larsen, L. F. S., Thybo, C., Stoustrup, J., Rasmussen, H., A method for online steady state energy minimization, with application to refrigeration systems. In 2004 43rd IEEE Conference on Decision and Control (CDC)(IEEE Cat. No. 04CH37601) (Vol. 5, pp. 4708-4713). (2004, December). IEEE.

[24] Kim, M. S., Shin, C. S., Kim, M. S., A study on the real time optimal control method for heat rejection pressure of a CO2 refrigeration system with an internal heat exchanger. International Journal of Refrigeration, 48 (2014), 87-99.

[25] Larsen, L. S., Thybo, C., Potential energy savings in refrigeration systems using optimal set-points. In Proceedings of the 2004 IEEE International Conference on Control Applications, 2004. (Vol. 1, pp. 701-704). (2004, September), IEEE.

[26] Larsen, L. F. S., Model based control of refrigeration systems. Department of Control Engineering, Aalborg University, (2006).

[27] Wang, Y., Niu, S., Fu, W., Sensitivity analysis and optimal design of a dual mechanical port idirectional flux-modulated machine. IEEE Transactions on Industrial Electronics, 65(1) (2018), 211-220.

[28] Fouladirad, M., Paroissin, C., Grall, A., Sensitivity of optimal replacement policies to lifetime parameter estimates. European Journal of Operational Research, 266(3) (2018), 963-975.

[29] Zafar, F., Cordero, A., Torregrosa, J., An efficient family of optimal eighth-order multiple root finders. Mathematics, 6(12) (2018), 310.

[30] Elaoud, S., Hadj-Taieb, L., Hadj-Taieb, E., Leak detection of hydrogen-natural gas mixtures in pipes using the characteristics method of specified time interval. J. Loss Prev. Process Industries 23 (2010), 637-645.

[31] Yu, M., Lin, J., Taylor series expansion scheme applied for solving population balance equation. Reviews in Chemical Engineering, 34(4) (2018), 561-594.

[32] Sarkhosh, S.Ch., Kiai, S. S., Bakar, M. A., Ziaeian, I., Fudziah, I., A new impulsional potential for a Paul ion trap. International Journal of Mass Spectrometry, 309 (2012), 63-69.

[33] Sarkhosh, S.Ch., Moameni, A., Nanoparticle Volume Fraction Optimization for Efficient Heat Transfer and Heat Flow Problems in a Nanofluid. Heat Transfer Research, (2018) 49(8).

[34] Tomai, S., Dovan, D., krjanc, I., Confidence-Interval-Fuzzy-Model-Based Indoor Localization. IEEE Transactions on Industrial Electronics, 66(3) (2019), 2015-2024.

[35] Lee, W. R., The confidence interval reported in this study appears to be an error. Brachytherapy, (2019).

[36] Cavenagh, N. J., Wright, L. K., The maximum, spectrum and supremum for critical set sizes in (0, 1)-matrices. arXiv preprint arXiv:1812.08282, (2018).

Click here to access/download
Supporting Information
Graphical Abstract.pdf

Click here to access/download
Supporting Information
Declaration of Interest Statement.docx
