# Supplementary material for: Controlling the pressure of hydrogen-natural gas mixture in an inclined pipeline
Source: PLoS One. 2020 Feb 27;15(2):e0228955. doi: 10.1371/journal.pone.0228955 (PMC7046196; doi:10.1371/journal.pone.0228955)
Supplement: Attachment — Submitted filename: Response to Reviewers.docx [file pone.0228955.s008.docx]

We warmly thank the very competent Editor and Reviewers for the time spent on reading the manuscript and for the valuable comments and suggestions. We have modified the manuscript accordingly, and the detailed corrections are listed below point by point.

1. We note the following previous request has not been addressed:

PLOS requires an ORCID iD for the corresponding author in Editorial Manager on papers submitted after December 6th, 2016. Please ensure that you have an ORCID iD and that it is validated in Editorial Manager. To do this, go to ‘Update my Information’ (in the upper left-hand corner of the main menu), and click on the Fetch/Validate link next to the ORCID field. This will take you to the ORCID site and allow you to create a new iD or authenticate a pre-existing iD in Editorial Manager. Please see the following video for instructions on linking an ORCID iD to your Editorial Manager account: <https://www.youtube.com/watch?v=_xcclfuvtxQ>

Answer: has been added as follows:

**sarkhosh seddighi chaharborj** <https://orcid.org/0000-0001-5894-6622>

2. In your Data Availability statement, you have not specified where the minimal data set underlying the results described in your manuscript can be found. PLOS defines a study's minimal data set as the underlying data used to reach the conclusions drawn in the manuscript and any additional data required to replicate the reported study findings in their entirety. All PLOS journals require that the minimal data set be made fully available. For more information about our data policy, please see http://journals.plos.org/plosone/s/data-availability.

Upon re-submitting your revised manuscript, please upload your study’s minimal underlying data set as either Supporting Information files or to a stable, public repository and include the relevant URLs, DOIs, or accession numbers within your revised cover letter. For a list of acceptable repositories, please see <http://journals.plos.org/plosone/s/data-availability#loc-recommended-repositories.> Any potentially identifying patient information must be fully anonymized.

Important: If there are ethical or legal restrictions to sharing your data publicly, please explain these restrictions in detail. Please see our guidelines for more information on what we consider unacceptable restrictions to publicly sharing data: <http://journals.plos.org/plosone/s/data-availability#loc-unacceptable-data-access-restrictions.> Note that it is not acceptable for the authors to be the sole named individuals responsible for ensuring data access.

We will update your Data Availability statement to reflect the information you provide in your cover letter.

Answer: We will share all supporting data’s have been used in this paper.

3. Please amend the title either on the online submission form or in your manuscript so that they are identical.

Answer: All title updated to this form:

“**Controlling the pressure of hydrogen-natural gas mixture in an inclined pipeline**“

4. Please upload a copy of Supporting Information Figures 1 - 10 which you refer to in your text on page 28.

Answer: All figures added in the page 28.

Journal Requirements:

1. Please ensure that your manuscript meets PLOS ONE's style requirements, including those for file naming. The PLOS ONE style templates can be found at http://www.journals.plos.org/plosone/s/file?id=wjVg/PLOSOne_formatting_sample_main_body.pdf and <http://www.journals.plos.org/plosone/s/file?id=ba62/PLOSOne_formatting_sample_title_authors_affiliations.pdf>

Answer: Carefully paper style checked.

2. We note that you have stated that you will provide repository information for your data at acceptance. Should your manuscript be accepted for publication, we will hold it until you provide the relevant accession numbers or DOIs necessary to access your data. If you wish to make changes to your Data Availability statement, please describe these changes in your cover letter and we will update your Data Availability statement to reflect the information you provide.

Answer: We didn’t used laboratory data or real data in our paper.

3. PLOS requires an ORCID iD for the corresponding author in Editorial Manager on papers submitted after December 6th, 2016. Please ensure that you have an ORCID iD and that it is validated in Editorial Manager. To do this, go to ‘Update my Information’ (in the upper left-hand corner of the main menu), and click on the Fetch/Validate link next to the ORCID field. This will take you to the ORCID site and allow you to create a new iD or authenticate a pre-existing iD in Editorial Manager. Please see the following video for instructions on linking an ORCID iD to your Editorial Manager account: <https://www.youtube.com/watch?v=_xcclfuvtxQ>

Answer: 1- sarkhosh seddighi chaharborj (<https://orcid.org/0000-0001-5894-6622)>

2- Norsarahaida Amin (https://orcid.org/0000-0002-0308-679X)

4. Please include captions for your Supporting Information files at the end of your manuscript, and update any in-text citations to match accordingly. Please see our Supporting Information guidelines for more information: <http://journals.plos.org/plosone/s/supporting-information>.

Answer: Has been done carefully.

5. We noticed you have some minor occurrence of overlapping text with the following previous publication(s), which needs to be addressed:

<http://dx.doi.org/10.1155/2015/510675>

<http://dx.doi.org/10.11648/j.acm.20150403.16>

<https://doi.org/10.1016/j.jlp.2017.05.003>

Answer: All 3 papers have been cited in the preset paper, but main paper is <https://doi.org/10.1016/j.jlp.2017.05.003>

And out results have been compared with this paper results.

References edited and updated as follows:

**References**

[1] Elaoud, S., Hadj-Taieb, E., Transient flow in pipelines of high-pressure hydrogen-natural gas mixtures. Int. J. Hydrogen Energy. 2008; 33 (18): 4824-4832.
[2] Chaczykowski, M., Transient flow in natural gas pipeline the effect of pipeline thermal model. Appl. Math. Model. 2010; 34: 1051-1067.
[3] Karney, B.W., Ruus, E., Charts for water hammer in pipelines resulting from valve closure from full opening only. Canadian Journal of Civil Engineering, 1985; 12(2): 241-264.
[4] Kim, H., Kim, S., Kim, Y., Kim, J., Optimization of Operation Parameters for Direct Spring Loaded Pressure Relief Valve in a Pipeline System. Journal of Pressure Vessel Technology. 2018; 140(5): 051603.
[5] Jalving, J., Zavala, V. M., An Optimization-Based State Estimation Framework for Large-Scale Natural Gas Networks. Industrial & Engineering Chemistry Research. 2018; 57(17): 5966-5979.
[6] Subani, N., Amin, N., Agaie, B.G., Leak detection of non-isothermal transient flow of hydrogennatural gas mixture. Journal of Loss Prevention in the Process Industries. 2017; 48: 244-253.
[7] Subani, N., Amin, N., Analysis of water hammer with different closing valve laws on transient flow of hydrogen-natural gas mixture. Abstr. Appl. Analysis. 2015; 2015: 1-12.
[8] Subani, N., Amin, N., Agaie, B.G., Hydrogen-natural gas mixture leak detection using reduced order modelling. Appl. Comput. Math. 2012; 4 (3): 135-144.
[9] Uilhoorn, F.E., Dynamic behaviour of non-isothermal compressible natural gases mixed with hydrogen in pipelines. Int. J. Hydrogen Energy. 2009; 34 (16): 6722-6729.
[10] Yu, T., Ding, Q., Wang, L., Zhu, D. Z., Shao, Y., Experimental study on air pressure variation in a horizontal pipe of single-stack drainage system. Water Science and Technology. 2019; 79(1): 114-125.
[11] Zaki, K., Imam, Y., El-Ansary, A., Optimizing the dynamic response of pressure reducing valves to transients in water networks. Journal of Water Supply: Research and Technology-Aqua. DOI: 102166/aqua.2019.121.
[12] Hennings, F., Anderson, L., Hoppmann, K., Turner, M., Koch, T., Controlling transient gas flow in complex pipeline intersection areas: 2019.
[13] Osiadacz, A. J., Chaczykowski, M., Optimization of Gas Networks Under Transient Conditions. In PSIG Annual Meeting. Pipeline Simulation Interest Group. DOI:10.1016/j.jngse.2018.03.014.
[14] Liu, B., Liu, X., Lu, C., Godbole, A., Michal, G., Teng, L., Decompression of hydrogennatural gas mixtures in high-pressure pipelines: CFD modelling using different equations of state. International Journal of Hydrogen Energy. 2019; 44(14): 7428-7437.
[15] Noeding, M., Tegethoff, W., Koehler, J., Zero Gradient Control for R-744 Refrigeration Cycles. International Journal of Refrigeration. DOI: 10.1016/j.ijrefrig.2019.05.041.
[16] Hu, L., Xue, F., Qin, Z., Shi, J., Qiao, W., Yang, W., Yang, T., Sliding mode extremum seeking control based on improved invasive weed optimization for MPPT in wind energy conversion system. Applied Energy. 2019; 248: 567-575.
[17] Larsen, L. F. S., Thybo, C., Stoustrup, J., Rasmussen, H., A method for online steady state energy minimization, with application to refrigeration systems. In 2004 43rd IEEE Conference on Decision and Control (CDC)(IEEE Cat. No. 04CH37601) (Vol. 5, pp. 4708-4713) (2004, December). IEEE.
[18] Zhang, W. J., Zhang, C. L., A correlation-free on-line optimal control method of heat rejection pressures in CO2 transcritical systems. International Journal of Refrigeration. 2011; 34(4): 844-850.
[19] Kim, M. S., Kim, M. S., An on-line control method of optimal heat rejection pressure for CO2 refrigeration cycle. In Proceedings of the 10th IIR Gustav Lorentzen Conference on Natural Refrigerants, Delft, The Netherlands, Paper No. GL-303; 2012.
[20] Elaoud, S., Hafsi, Z., & Hadj-Taieb, L. (2017). Numerical modelling of hydrogen-natural gas mixtures flows in looped networks. Journal of Petroleum Science and Engineering, 159, 532-541.
[21] Maxey, W. A., Fracture initiation, propagation, and arrest. In Proc. of Symposium on Line Pipe Research; 1974.
[22] Jensen, J. B., Skogestad, S., Optimal operation of simple refrigeration cycles: Part I: Degrees of freedom and optimality of sub-cooling. Computers & chemical engineering. 2012; 31(5-6):712-721.
[23] Koeln, J. P., Alleyne, A. G., Optimal subcooling in vapor compression systems via extremum seeking control: Theory and experiments. International Journal of Refrigeration. 2014; 43:14-25.
[24] Larsen, L. F. S., Thybo, C., Stoustrup, J., Rasmussen, H., A method for online steady state energy minimization, with application to refrigeration systems. In 2004 43rd IEEE Conference on Decision and Control IEEE. 2004; 5: 4708-4713.
[25] Kim, M. S., Shin, C. S., Kim, M. S., A study on the real time optimal control method for heat rejection pressure of a CO2 refrigeration system with an internal heat exchanger. International Journal of Refrigeration. 2014; 48: 87-99.
[26] Larsen, L. S., Thybo, C., Potential energy savings in refrigeration systems using optimal set-points. In Proceedings of the 2004 IEEE International Conference on Control Applications. IEEE. 2004; 1: 701-704.
[27] Larsen, L. F. S., Model based control of refrigeration systems. Department of Control Engineering, Aalborg University; 2006.
[28] Wang, Y., Niu, S., Fu, W., Sensitivity analysis and optimal design of a dual mechanical port idirectional flux-modulated machine. IEEE Transactions on Industrial Electronics. 2018; 65(1):211-220.
[29] Fouladirad, M., Paroissin, C., Grall, A., Sensitivity of optimal replacement policies to lifetime parameter estimates. European Journal of Operational Research. 2018; 266(3):963-975.
[30] Zafar, F., Cordero, A., Torregrosa, J., An efficient family of optimal eighth-order multiple root finders. Mathematics. 2018; 6(12): 310-326.
[31] Elaoud, S., Hadj-Taieb, L., Hadj-Taieb, E., Leak detection of hydrogen-natural gas mixtures in pipes using the characteristics method of specified time interval. J. Loss Prev. Process Industries. 2010; 23: 637-645.
[32] Yu, M., Lin, J., Taylor series expansion scheme applied for solving population balance equation. Reviews in Chemical Engineering. 2018; 34(4): 561-594.
[33] Sarkhosh, S.Ch., Kiai, S. S., Bakar, M. A., Ziaeian, I., Fudziah, I., A new impulsional potential for a Paul ion trap. International Journal of Mass Spectrometry. 2012; 309: 63-69.
[34] Sarkhosh, S.Ch., Moameni, A., Nanoparticle Volume Fraction Optimization for Efficient Heat Transfer and Heat Flow Problems in a Nanofluid. Heat Transfer Research. 2018; 49(8):761-771.
[35] Tomai, S., Dovan, D., krjanc, I., Confidence-Interval-Fuzzy-Model-Based Indoor Localization. IEEE Transactions on Industrial Electronics. 2019; 66(3): 2015-2024.
[36] Lee, W. R., The confidence interval reported in this study appears to be an error. Brachytherapy. 2019; 18(3); 426.
[37] Cavenagh, N. J., Wright, L. K., The maximum, spectrum and supremum for critical set sizes in (0, 1)-matrices. 2019; 27(8): 522-536.

**Supporting information’s have been added as follows:**

- **Captions for your Supporting Information files**

**Caption of figures:**

**Fig. 1:** The system consists of a simple inclined pipeline with a reservoir upstream and a valve downstream of the pipeline.

**Fig. 2:** Density as a function of the pressure for different values of $\phi$.

**Fig.3:** Celerity pressure wave as a function of the pressure for different values of $\phi.$

**Fig.4:** Permanent regime pressure distribution along the pipe (equations (13) and (14)) for different mass ratio $\phi=0, 0.5, 1$.

**Fig.5:** Transient pressure distribution for the rapid closure valve at the downstream end of the pipe for different values of mass ratio $\phi$ and $\theta=0$ [8, 30].

**Fig.6:** Transient pressure distribution for the rapid closure valve at the downstream end of the pipe. (a): for different values of $\theta=0,\pi/6,\pi/4,\pi/3$ and mass ratio $\phi=0.5$; (b): for different values of mass ratio $\phi=0, 0.5, 1$ and $\theta=0$; (c): for different values of permanent pressure $P_{0}=30, 60$ and $\theta=0$, $\phi=0.5$.

**Fig.7:** Variation of optimal pressure using equation (21); (a): as a function of the initial pressure $P_{0}$, (b): as a function of the mass ratio $\phi$.

**Fig.8:** Variation of optimal pressure using equation (22); (a): as a function of the initial pressure $P_{0}$, (b): as a function of the mass ratio $\phi$.

**Fig.9:** Variation of optimal mass ratio as a function of pressure with different initial pressure by using, (a): equation (5) and (b): equation (7).

**Fig.10:** Transient pressure of hydrogen natural gas mixture for isothermal flow when leakage occurs at $X_{L}=L/3$ in horizontal pipeline; red: $\phi=0.25$ and black:$\phi=0.5$.

**Caption of Tables:**

**Table 1:** Hydrogen properties in working conditions, $P=35$ bar and T = 15${}^{^{\circ}}C$=288 K [6,30].

**Table 2:** Parameters used for the simulation [6, 30].

**Table 3:** Values of coefficients $a_{0}, a_{1}, a_{2}$ and $b_{0}, b_{1}, b_{2}$ for various $\phi$ in Eq. (11).

Reviewers' comments:

Reviewer's Responses to Questions

**Comments to the Author**

We warmly thank the very competent Reviewers for the time spent on reading the manuscript and for the valuable comments and suggestions. We have modified the manuscript accordingly, and the detailed corrections are listed below point by point.

1. Is the manuscript technically sound, and do the data support the conclusions?

The manuscript must describe a technically sound piece of scientific research with data that supports the conclusions. Experiments must have been conducted rigorously, with appropriate controls, replication, and sample sizes. The conclusions must be drawn appropriately based on the data presented.

Reviewer #1: Yes

Reviewer #2: Partly

Answer: In this paper, we didn’t used experimental data, just we compared our results with previous published papers results.

2. Has the statistical analysis been performed appropriately and rigorously?

Reviewer #1: Yes

Reviewer #2: I Don't Know

Answer: All supporting data’s will share if our paper accepted for publication.

3. Have the authors made all data underlying the findings in their manuscript fully available?

The [PLOS Data policy](http://www.plosone.org/static/policies.action#sharing) requires authors to make all data underlying the findings described in their manuscript fully available without restriction, with rare exception (please refer to the Data Availability Statement in the manuscript PDF file). The data should be provided as part of the manuscript or its supporting information, or deposited to a public repository. For example, in addition to summary statistics, the data points behind means, medians and variance measures should be available. If there are restrictions on publicly sharing data—e.g. participant privacy or use of data from a third party—those must be specified.

Reviewer #1: Yes

Reviewer #2: Yes

4. Is the manuscript presented in an intelligible fashion and written in standard English?

PLOS ONE does not copyedit accepted manuscripts, so the language in submitted articles must be clear, correct, and unambiguous. Any typographical or grammatical errors should be corrected at revision, so please note any specific errors here.

Reviewer #1: Yes

Reviewer #2: No

Answer: Thank you very much because of good comments. Papers checked carefully according the referee attached and we did all comments as them asking.

5. Review Comments to the Author

Please use the space provided to explain your answers to the questions above. You may also include additional comments for the author, including concerns about dual publication, research ethics, or publication ethics. (Please upload your review as an attachment if it exceeds 20,000 characters)

Reviewer #1: 1. Turnitin results showed a high similarity index (29%) for this paper (exclude quotes on, exclude bibliography on and exclude matches < 1%). Please revise first.

Answer: Checked for similarity.

2. A method called Zero Gradient Control (ZGC) was said to control the optimal transient pressure. However, it is not clear the performance of the transient pressure for regulatory or servo problem.

Answer: Thank you for nice comment. New results have been added as follows,

Fig.(10) shows the transient pressure of hydrogen natural gas mixture for isothermal flow when leakage occurs at $X_{L}=L/3$ in horizontal pipeline; red is transient pressure for gas mixture $\phi=0.25$ and black is transient pressure for gas mixture$\phi=0.5$. As seen from this figure for $\nabla\phi=0.25-0=0.25$ we have $\nabla P=8.708$ and $\nabla\phi=0.5-0=0.5$ we have $\nabla P=10.031$ ($P$ is transient pressure).

**Fig.10:** Transient pressure of hydrogen natural gas mixture for isothermal flow when leakage occurs at $X_{L}=L/3$ in horizontal pipeline; red: $\phi=0.25$ and black:$\phi=0.5$.

3. Please state clearly whether the optimal set point is for controller equipment such as PID controller, or else with different meaning.

Answer: Thank you for nice comments.

Fig.(10) shows the transient pressure of hydrogen natural gas mixture for isothermal flow when leakage occurs at $X_{L}=L/3$ in horizontal pipeline; red is transient pressure for gas mixture $\phi=0.25$ and black is transient pressure for gas mixture$\phi=0.5$. As seen from this figure for $\nabla\phi=0.25-0=0.25$ we have $\nabla P=8.708$ and $\nabla\phi=0.5-0=0.5$ we have $\nabla P=10.031$ ($P$ is transient pressure).

**Fig.10:** Transient pressure of hydrogen natural gas mixture for isothermal flow when leakage occurs at $X_{L}=L/3$ in horizontal pipeline; red: $\phi=0.25$ and black:$\phi=0.5$.

Reviewer #2: Please put more effort in correct English. Furthermore, variables are not named uniform and often units are missing etc. In general you have written a nice piece of work, but it cannot be published yet in its current form. See attachment for further comments

Answer: Has been checked carefully according the attached comments.

6. PLOS authors have the option to publish the peer review history of their article ([what does this mean?](https://journals.plos.org/plosone/s/editorial-and-peer-review-process#loc-peer-review-history)). If published, this will include your full peer review and any attached files.

If you choose “no”, your identity will remain anonymous but your review may still be made public.

**Do you want your identity to be public for this peer review?** For information about this choice, including consent withdrawal, please see our [Privacy Policy](https://www.plos.org/privacy-policy).

Reviewer #1: No

Reviewer #2: No
